# The Catalytically Inactive KDM5B-NTT Isoform Associates with Chromatin and Increases H3K4 Trimethylation in Breast Cancer Cells

**DOI:** 10.3390/ijms27198774

**Published:** 2026-09-30

**Authors:** Valeria Manzini, Elena Di Nisio, Alessandro Piana, Valerio Perticaroli, Italia Anna Asteriti, Federica Polverino, Rodolfo Negri, Valerio Licursi

**Affiliations:** 1 Department of Biology and Biotechnologies “C. Darwin”, Sapienza University of Rome, 00185 Rome, Italy; 2 Institute of Molecular Biology and Pathology (IBPM), National Research Council (CNR) of Italy, 00185 Rome, Italy

**Keywords:** KDM5B, protein isoforms, MCF7, cancer epigenetics, histone modifications, cell migration, cell invasion

## Abstract

Histone lysine demethylases are mainly known as transcriptional regulators for their demethylase activity, but catalysis-independent functions are increasingly recognized. This work focuses on lysine-specific demethylase 5B (KDM5B), targeting H3K4me3. The canonical functional isoform is PLU-1. A catalytically inactive N-terminally truncated isoform (NTT) was previously identified in human cell lines. NTT lacks the Jumonji N-terminal (JmjN) domain, responsible for demethylase activity, and part of the AT-Rich Interaction Domain (ARID), but retains the plant homeodomains (PHDs) involved in chromatin interaction. The study aimed to investigate NTT chromatin association, its potential regulatory role, and its phenotypic effects. NTT-FLAG was overexpressed in MCF7 cells, using PLU-1-FLAG and empty vector for comparison. Chromatin association and H3K4me3 distribution were analyzed by Cleavage Under Targets and Release Using Nuclease (CUT&RUN), while migration and invasion were assessed by wound-healing, live-imaging microscopy, and invasion assays. NTT-FLAG and PLU-1-FLAG displayed similar genomic localization, preferentially associating with active promoters enriched in H3K4me3. Both isoforms increased promoter-associated H3K4me3, suggesting that chromatin binding alone is insufficient to promote demethylation. Functionally, NTT overexpression was associated with increased directional migration and invasive capacity compared with PLU-1. Overall, these findings support a catalysis-independent regulatory role for NTT and suggest a model in which chromatin interaction is driven by PHD domains, while co-interactor availability may be required for proper PLU-1 demethylase activity.

## 1. Introduction

It is well known that the dynamicity and complexity of the epigenetic code contribute to fine-tuning gene expression by regulating chromatin accessibility to protein complexes, including chromatin remodelers and transcription factors [1,2]. Among all the epigenetic modifications in this complex regulatory system, histone lysine methylation can occur at three levels: mono-, di-, and tri-methylation. Histone lysine methylation modulates a variety of biological processes, including transcription, and provides both activator and repressive signals depending on the lysine residue [2,3,4,5,6]. Normally, trimethylations on K4, K36, and K79 of histone 3 (H3) tend to promote an active transcriptional state, while H3K9me2/3 and H3K27me3 are associated with transcription repression. However, the exact outcome of methylation on gene expression depends on the precise equilibrium between signals arising from the co-presence of various epigenetic modifications and their relative amounts, the genomic localization, and the dynamic actions of writers, erasers, and readers [7].

In humans, more than 100 lysine methyltransferases (KTMs) act on histone and non-histone proteins [5]. These enzymes are the writers, and their activity is counterbalanced by lysine demethylases (KDMs), which are considered both readers and erasers [7]. KDMs are grouped into two families: the KDM1, or Lysine Specific histone Demethylases (LSD), and the Jumonji Histone Demethylases (JHDMs), which include subfamilies from KDM-2 to KDM-7 [2,3,6]. In general, JHDMs are highly specific enzymes that act on specific lysine residues and their methylation state. Indeed, the H3K4me2/3 is recognized and removed by the KDM5 subfamily, which includes KDM5A, KDM5B, KDM5C and KDM5D [8]. Since H3K4me3 is a marker of open, active chromatin, mainly localized to promoter regions, KDM5s mainly act as transcriptional repressors. However, it could also promote canonical transcription by removing spurious transcription start sites within the gene ORF or by favoring the accumulation of H3K4me1 on enhancer regions, a marker of active enhancers [9,10].

KDM5s control a variety of processes during development and cellular differentiation [7], and are involved in tumor development and progression [10,11,12,13], as well as chemoresistance [14,15,16]. Specifically, several cancer types are characterized by elevated KDM5B expression, likely due to increased gene copy number, whereas in most healthy adult tissues, it is expressed at modest levels [13,17,18]. Indeed, a consistent up-regulation of KDM5B was observed in tumors of breast [19], prostate [20], melanoma [21,22], bladder and lungs [23]. However, the role of KDM5B in tumors could be dual. In melanoma, two distinct co-existing cell populations were described: the majority of cells expressed low levels of KDM5B and exhibited faster proliferation and colonisation, while a small population produced higher levels of this protein and had stem-like features, likely creating a staminal niche for melanoma maintenance [21,22,24]. In breast cancer, a different behavior of KDM5B was observed in different subtypes of cancer. Luminal breast cancer cell lines, where KDM5B acts as an oncogene, are particularly sensitive to KDM5B inhibition [15,16,17,25,26,27,28]. In this model, KDM5B represses several tumor suppressor genes, including *BRCA1* and *CAV1*. In basal triple-negative breast cancer cell lines, which are less responsive to KDM5B silencing, the protein appears to repress specific tumor-related phenotypes, including cell proliferation, migration, and invasion [7,17,25,27,29,30].

In recent years, a variety of demethylase-independent roles have been described for KDMs, including enzymatically distinct activities and catalytically independent roles [31]. KDM5s emerged in their role as scaffolds, bringing several protein complexes to chromatin and directly regulating gene expression and chromatin folding without demethylating histone lysines. The versatility of the KDMs may be due to their multi-domain composition. In particular, the main domains of KDM5s are: AT-Rich Interaction Domain (ARID) for DNA binding, a plant homeodomain 1 (PHD1) for recognising H3K4me0, a plant homeodomain 3 (PHD3) for recognising H3K4me3, and Jumonji N-terminal (JmjN) and C-terminal (JmjC) required for demethylase activity [9,25].

Interestingly, the development of the ΔARID mouse model demonstrated that a catalytically inactive KDM5B can be compatible with life. In fact, these mice expressed a protein lacking the entire ARID domain and a few amino acids of the JmjN domain, rendering it unable to demethylate histone lysines. Previous attempts to generate KDM5B knockout models failed due to early lethality. Given these facts, the catalytic activity of KDM5B may be replaced by other demethylases, but its non-catalytic roles are required for embryogenesis and development [32].

In humans, several KDM5B isoforms derived from different transcripts were described [33]. Among them, the completely functional PLU-1 isoform is considered the most abundant and the canonical one. In our previous work, an uncharacterized human KDM5B isoform was described. It was called NTT (N-Terminal Truncated) for its N-terminal truncation that depletes the entire JmjN domain and part of the ARID [33]. Even if catalytically inactive, this protein likely retains chromatin-interacting ability through its PHDs, potentially competing with canonical PLU-1 binding. NTT contains an extra exon-6, which is absent in PLU-1, and is expressed at low levels across a variety of human cancer cell lines and tissues, where it can eventually accumulate thanks to remarkable protein stability. The ectopic overexpression of this isoform in the MCF7 luminal breast cancer cell line increased global H3K4me3 levels in bulk chromatin and influenced the expression of genes involved in specific biological processes, including immune response and tumor-related phenotypes. The proposed interpretation of these data was that NTT may bind the same target sites as PLU-1, interfering with the demethylase activity of functional isoforms and acting as a dominant-negative competitor for PLU-1.

The present work aimed to demonstrate the association of NTT with chromatin and characterize its effects on H3K4me3 on genomic targets. Moreover, the effects of NTT overexpression on a selected tumor-related phenotype, such as cell migration and invasion, were described.

## 2. Results

### 2.1. Genomic Localization of Overexpressed NTT and PLU-1 Isoforms

**Figure 1:**
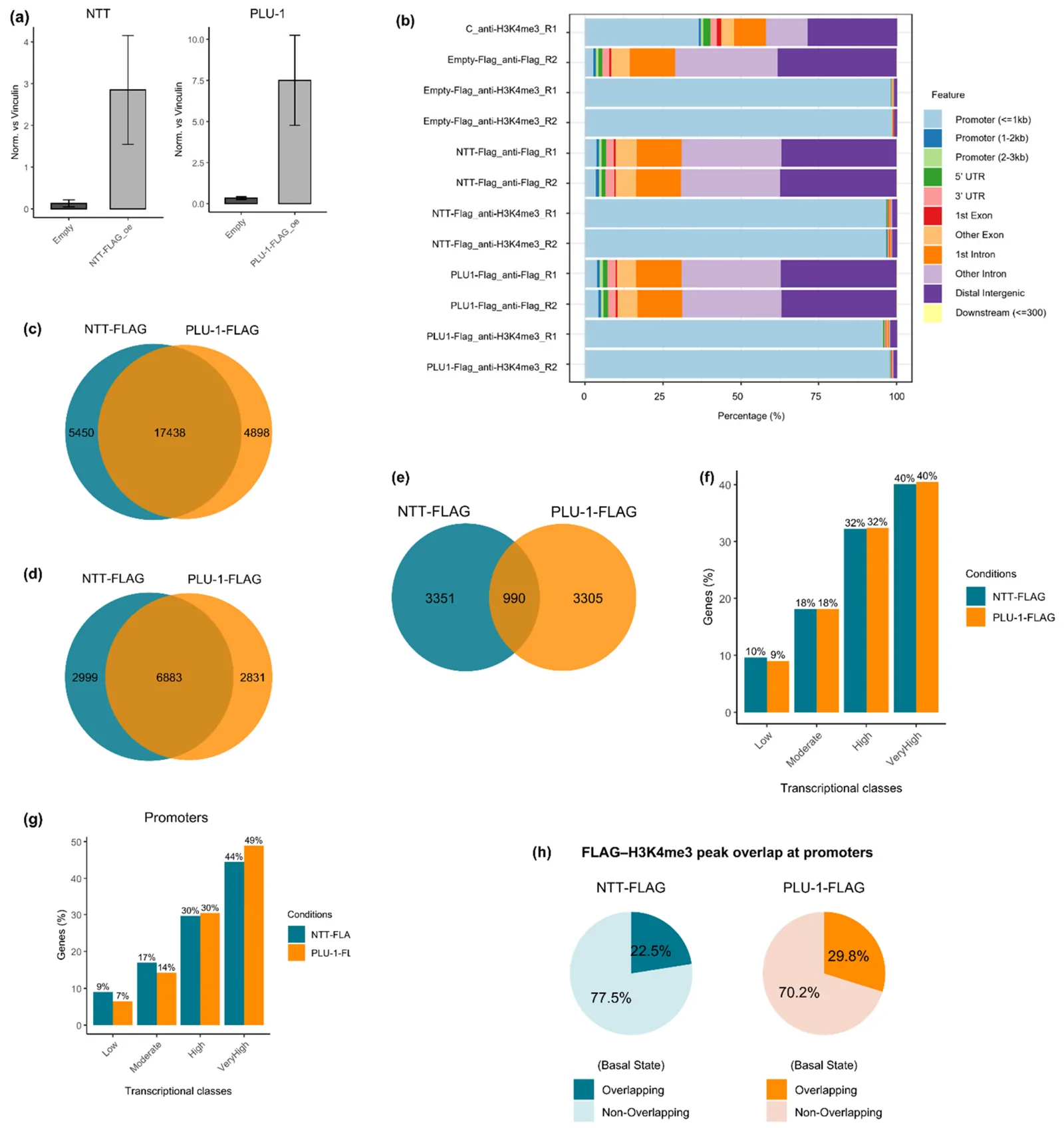
Genome-wide binding profiles of overexpressed N-Terminal Truncated (NTT)-FLAG and PLU-1-FLAG in MCF7 cells. The genomic distribution and transcriptional context of the two isoforms are reported. (**a**) Western blot analysis confirming the overexpression of the FLAG-tagged NTT and PLU-1 isoforms in MCF7 cells. Protein expression levels in cells transfected with the empty vector were compared to those overexpressing NTT-FLAG or PLU-1-FLAG in three independent analyses. Data are presented as mean ± standard error of the mean (SEM). Protein levels were normalized to vinculin as an endogenous reference; (**b**) Genomic feature distribution of binding peaks identified by CUT&RUN in MCF7 cells. Bar charts illustrate the percentage of peaks mapping to distinct genomic regions for each sample and biological replicate (R1, R2); (**c**) Venn diagram illustrating the overlap of all genes containing at least one NTT-FLAG (teal) or PLU-1 FLAG (orange) binding site; (**d**) Venn diagram depicting the overlap of genes nearest to distal intergenic regions containing NTT-FLAG (teal) or PLU-1 FLAG (orange) binding sites; (**e**) Venn diagram showing the overlap of gene containing NTT-FLAG (teal) or PLU-1 FLAG (orange) binding sites in their promoters; (**f**) percentage of genes harboring FLAG peaks across their entire length (gene body), stratified by transcriptional class for NTT-FLAG (teal) and PLU-1 FLAG (orange); (**g**) percentage of genes exhibiting FLAG peaks at their promoters, stratified by transcriptional class for NTT-FLAG (teal) and PLU-1 FLAG (orange); (**h**) pie charts detailing all FLAG peaks localized at promoters. Dark shades denote an overlap (direct or proximal) between FLAG-bound regions and basal H3K4me3 marks (in empty condition), whereas light shades indicate the absence of overlap.

To test the potential association of KDM5B-NTT with chromatin, the Cleavage Under Target & Release Using Nuclease (CUT&RUN) methodology was applied to the MCF-7 breast cancer cell line. In particular, NTT was tagged with a FLAG and overexpressed in cells via transfection with a plasmid vector (see Section 4). As a comparison, the overexpression of the FLAG-tagged canonical PLU-1 isoform was used together with a control represented by the empty-FLAG (E)-vector-transfected cells (Figure 1a). For the CUT&RUN experiments, an anti-FLAG antibody allowed the capture of both FLAG-tagged NTT and PLU-1 on chromatin, whilst the anti-H3K4me3 revealed H3K4me3 changes across the analyzed conditions. The anti-H3K4me3 antibody validated the success of the assay, showing a low background signal and the expected enrichment at transcription start sites (TSSs) and promoters (Figure 1b and Figure S1a,b). Consistently, pairwise correlation heatmaps showed high reproducibility across replicates within each condition (Figure S1c). The anti-FLAG signal, referred to as the empty-FLAG condition, was distributed across the genome in a noise-like manner, as expected for the absence of chromatin-binding proteins (Figure S1c). Regarding the signal distribution of overexpressed NTT- and PLU-1-FLAG, a discrete peak was observed around the TSS and within the promoter regions for both, with smaller defined peaks distributed in other areas, including non-coding elements (introns, distal intergenic) (Figure 1b and Figure S1b). Moreover, several peaks rarely perfectly coincided between the two replicates in both NTT and PLU-1 conditions.

Considering all the genes containing at least one peak of FLAG-tagged protein within their length, 22888 genes were targeted by NTT-FLAG and 22336 genes were bound by PLU-1-FLAG (Figure 1c). In intergenic regions, NTT-FLAG peaks were detected in 9882 distal sites, whereas PLU-1-FLAG targeted 9714 (Figure 1d). Interestingly, the isoforms shared more than 50% of their target genes (62.7% for all genes, 54.14% for distal intergenic sites). Moreover, 4341 genes contained NTT-FLAG peaks on their promoters and 4295 PLU-1-FLAG peaks; around 13% of the involved promoters were bound by both isoforms (Figure 1e). The presence of common targets may suggest shared mechanisms of target recognition and chromatin interaction between the NTT and PLU-1 isoforms.

To better understand the association criteria for the two proteins, their targets were classified according to their functional state. Mean transcriptional levels of protein-coding genes were obtained from RNA-seq of Empty-vector-treated cells [33] and were used to define four classes according to quartile distribution, so that each class contained 25% of the E population: Low, Moderate, High, Very High (Figure S1d). Then, the FLAG target genes were assigned to the defined classes, with about 20% unclassified, due to undetectable expression (Figure S1e). Both NTT and PLU-1 isoforms showed a clear tendency to associate with efficiently transcribed genes (Figure 1f). This tendency even increased when only the genes containing at least one FLAG binding site on their promoters were considered (Figure 1g).

The association of NTT and PLU-1 with chromatin may be influenced by the presence of promoter-associated H3K4me3 peaks, which, as shown in Figure S1e, are increasingly enriched at promoters of High and Very-High class genes. Indeed, peaks of FLAG-tagged NTT and PLU-1 are preferentially localized around the Transcription Start Site (TSS), with potential overlap with H3K4me3 peaks (Figure S1f). This overlap is negligible at the global level (below 4%, Figure S1g), as expected given that H3K4me3 is predominantly localized at promoters. However, it becomes more relevant in promoter regions, where 22.5% of NTT peaks and 29.8% of PLU-1 peaks coincide with or are adjacent to H3K4me3 peaks (Figure 1h). Our hypothesis implies that both NTT and PLU-1 may establish weak interactions with unmethylated H3K4 by PHD1, but when trimethylation is present, especially on promoters, PHD3 is recruited, stabilizing the interaction. This supports the previous assumption, suggesting that PHD3 is a major driver of PLU-1 chromatin interaction [25].

### 2.2. Effects of Overexpressed NTT and PLU-1 on H3K4 Trimethylation

**Figure 2:**
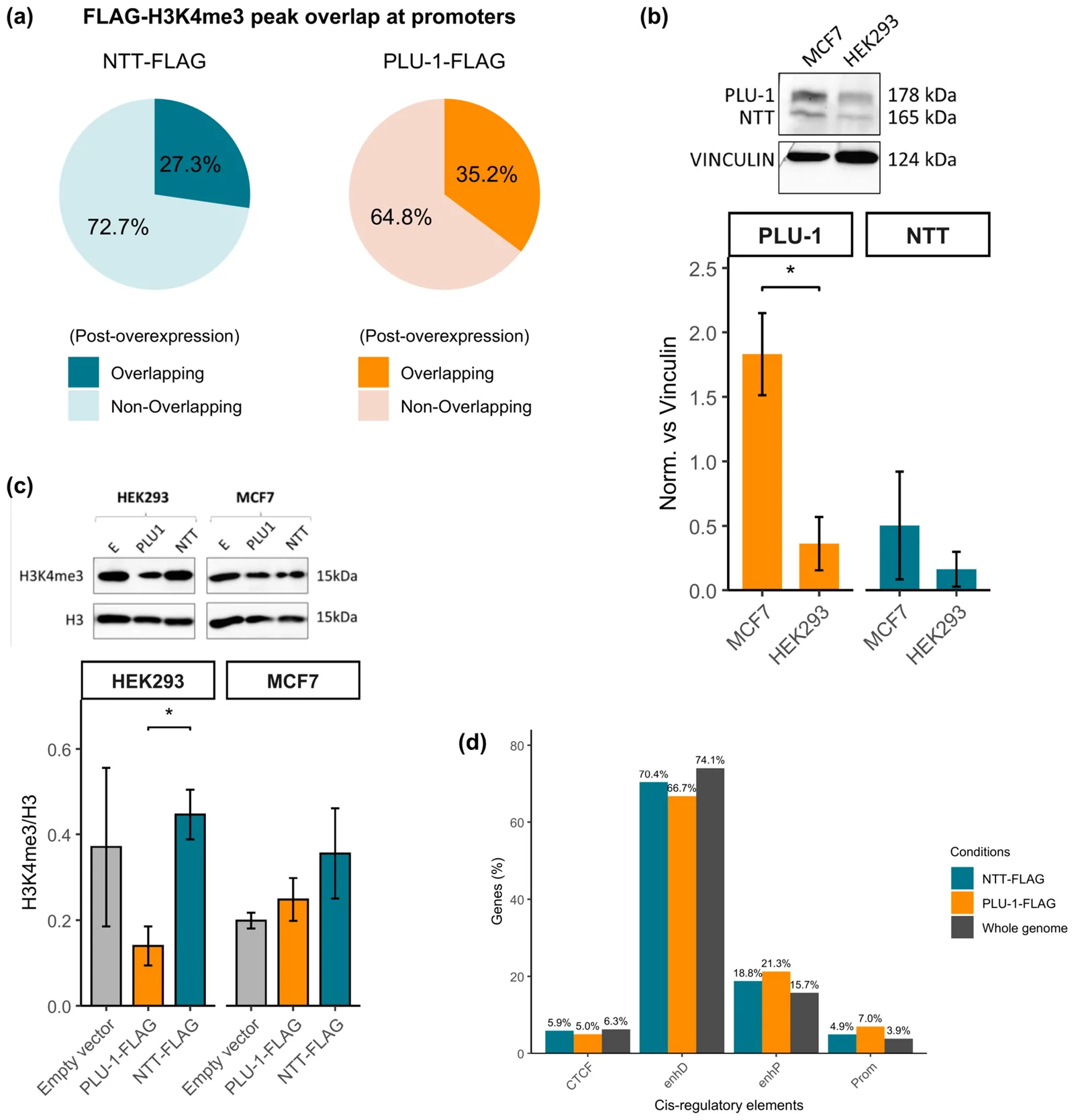
Impact of NTT-FLAG and PLU-1-FLAG overexpression on H3K4me3 levels and characterization of their binding at chromatin regulatory elements (cCREs). (**a**) Pie charts detailing all FLAG peaks localized at promoters upon overexpression of NTT-FLAG (teal) or PLU-1-FLAG (orange). Dark shades denote direct or proximal overlap between FLAG- interacting regions and H3K4me3 marks following overexpression of the two isoforms, whereas light shades indicate the absence of the overlap. (**b**) Protein levels of endogenous KDM5B isoforms (NTT, teal; PLU-1, orange) in untreated MCF7 and HEK293 cell lines, assessed by Western blot and normalized to vinculin. Each panel corresponds to one isoform, with cell lines displayed on the x-axis. Data are presented as mean ± SEM of three independent experiments. (**c**) Western blot quantification of global H3K4me3 levels, expressed as the H3K4me3/H3 ratio, in HEK293 and MCF7 cells under the indicated conditions. Each panel corresponds to one cell line, with experimental conditions displayed on the x-axis. Data are presented as mean ± SEM of three independent experiments. (**d**) Genomic distribution of FLAG-tagged isoform binding sites (NTT-FLAG, teal; PLU-1-FLAG, orange) classified as cCREs according to ENCODE annotation, across the following categories: CTCF-binding regions (CTCF), distal enhancers (enhD), proximal enhancers (enhP), and promoters (Prom). * *p* ≤ 0.05.

Next, changes in the presence of H3K4me3 at genes and promoters bound by NTT-FLAG and PLU-1-FLAG were analyzed. Interestingly, after overexpression of both KDM5B isoforms, an overall increase in H3K4me3 at promoters was observed (Figure 2a and Figure S2a). Specifically, the fraction of promoters bound by NTT-FLAG that showed H3K4me3 presence was 22.5% in E-vector-treated cells (Figure 1h) and rose to 27.3% after NTT-FLAG overexpression (Figure 2a). Similarly, for PLU-1-FLAG, this percentage changed from 29.8% in the basal condition (Figure 1h) to 35.2% upon overexpression (Figure 2a). This effect was expected for NTT, which, being catalytically inactive, could act as a dominant-negative competitor for PLU-1 and/or other class-5 histone demethylases, thereby protecting H3K4me3 sites from demethylase activity. However, it was less expected for PLU-1, which is an active histone demethylase. One possible explanation is that the catalytic activity of the FLAG-tagged version of the protein was impaired, although the purified enzyme has been shown to be efficient in vitro [34]. Alternatively, the association with H3K4me3 may not, per se, be sufficient to drive demethylation activity, and the presence of corepressor partners may be required. These co-interactors could become limiting for the action of ectopically expressed PLU-1 when a substantial amount of the endogenous PLU-1 is present. The implication could be that the majority of co-interactors are engaged in complexes with endogenous PLU-1, while the ectopically expressed protein may be unable to recruit free co-interactors to stimulate its activity.

To verify this hypothesis, we overexpressed NTT-FLAG and PLU-1-FLAG in the HEK-293 cell line, which has a significantly lower level (*p*_value = 0.024) of the endogenous PLU-1 compared to the MCF7 cells (Figure 2b), and analyzed the effects of their overexpression on H3K4me3 levels in the two cell lines (Figure 2c). While in the MCF7 cells no significant difference in the trimethylation level was evident between the cells transfected with the two isoforms, the HEK-293 cells transfected with PLU-1 FLAG showed significantly reduced levels (*p*_value = 0.016) of H3K4 trimethylation as compared with those transfected with NTT-FLAG. This result confirms that, in the context of reduced endogenous PLU-1, the ectopically expressed protein can reduce H3K4me3 levels, contrary to the truncated version.

Then, the occupancy of NTT-FLAG and PLU-1-FLAG on regulatory genomic regions was assessed, using the Candidate Cis-Regulatory Elements (cCREs) data from ENCODE as a reference. The dataset includes distal enhancers (EnhD), proximal enhancers (EnhP), promoters (Prom), and CTCF-binding regions (CTCF). Making the merge of coordinates referred to CREs and NTT-FLAG or PLU-1-FLAG binding peaks, 19% of the peaks were successfully classified, as expected, since the regulatory regions are a small fraction of the entire genome (Figure S2b). Both NTT-FLAG and PLU-1-FLAG were enriched at promoters and enhancers, confirming a preferential association with active loci (Figure 2d). Conversely, the fraction of peaks overlapping with CTCF-binding elements was lower than expected, particularly for PLU1-FLAG, although this difference did not reach statistical significance: endogenous KDM5B may already be bound to canonical CTCF regions, limiting access of the exogenously expressed FLAG-tagged isoforms to these sites and favoring their recruitment to alternative genomic locations. When H3K4me3 occupancy was examined across the same cCREs (Figure S2c), most regulatory regions lacked this modification in the basal state, and a small percentage acquired it after overexpression, particularly the enhancers after NTT-FLAG overexpression. Interestingly, the CTCF-interacting regions were the least affected by this increase, particularly in the case of PLU-1-FLAG overexpression, supporting the hypothesis that they are primarily occupied by endogenous KDM5B.

### 2.3. Effects of NTT Localization on H3K4me3 of Differentially Expressed Genes

At least 123 differentially expressed genes (DEGs) were previously identified in NTT-FLAG-overexpressing MCF7 cells compared with E-vector-treated cells [33]. All the methodological and statistical details regarding RNA-seq and DEG have been previously published in Di Nisio et al. [33]. While the previously established transcriptomic data characterized changes in the expression profile after NTT-FLAG overexpression, the present study builds upon those findings to introduce novel chromatin-binding data generated via CUT&RUN methodology. To investigate whether NTT-FLAG physically localizes to these deregulated genes, and whether PLU-1-FLAG shares a similar target recognition pattern, the genomic association of both NTT-FLAG and PLU-1-FLAG with these specific DEGs was evaluated. In particular, NTT-FLAG is associated with 32 genes, while PLU1-FLAG bound 28 genes, sharing 22 DEGs (Table 1). The list is enriched in genes that are functionally involved in the interferon response and immune pathways (summarized in Table S1). In all cases, the binding was spread across the entire transcription unit with multiple peaks. Next, to explore how the presence of these isoforms, particularly the binding of NTT-FLAG, might correlate with potential epigenetic alterations in transcriptional regulation, H3K4me3 at promoters were examined in all the specific cases. All the genes had well-defined H3K4me3 peaks at their promoters, except for *AZGP-1*, *TAGLN*, *CCL-5*, and *IL-32*. Interestingly, four genes associated with NTT-FLAG (*RSAD-2*, *OAS-2*, *IFIT-1*, *HLA-B*) and five genes associated with both isoforms (*SAMD-9*, *IFIT-3*, *OASL*, *CSF-1*, *TAP-2*) showed an evident extension in length of the H3K4me3 peak on promoters in NTT-FLAG or PLU1-FLAG-overexpressing cells compared with the Empty-vector condition (Figure S3a,b). In all the other cases, the trimethylation state of H3K4 appeared unchanged.

### 2.4. Phenotypical Analysis of Cell Migration and Invasion

**Figure 3:**
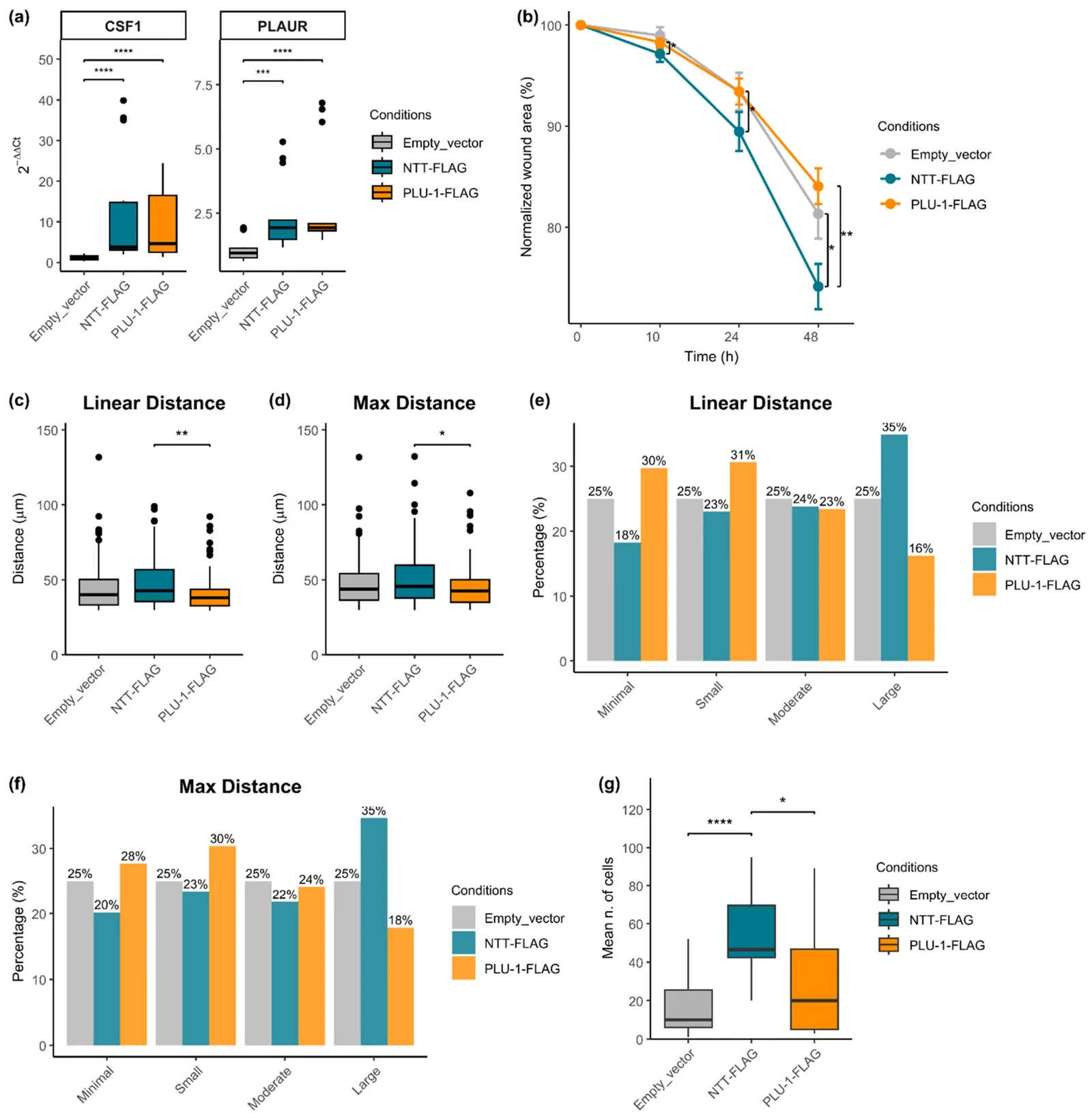
Effects of KDM5B isoform overexpression on migration and invasion in MCF7 cells. Overexpression of NTT-FLAG and PLU-1-FLAG differentially modulates the expression of motility-related genes and impacts cell migration and invasion capacity. (**a**) Transcript levels of *CSF-1* and *PLAUR* in the indicated conditions in MCF7 cells, assessed by RT-qPCR (biological quadruplicate) and expressed as 2^−ΔΔCt^. Statistical comparisons were performed using a Tukey post hoc test. For *CSF-1*: Empty vs. NTT-FLAG *p*-adj = 4.18 × 10^−6^, Empty vs. PLU-1-FLAG *p*-adj = 1.03 × 10^−5^, NTT-FLAG vs. PLU-1-FLAG *p*-adj = 0.982. For *PLAUR*: Empty vs. NTT-FLAG *p*-adj = 3.02 × 10^−4^, Empty vs. PLU-1-FLAG *p*-adj = 2.6 × 10^−5^, NTT-FLAG vs. PLU-1-FLAG *p*-adj = 0.722. (**b**) Wound closure quantification assessed by wound healing assay (biological triplicate). The wound area at each time point is normalized to the corresponding area at time 0 h within the same condition, thereby expressing the reduction in gap area as a percentage change rather than absolute area. Statistical analysis was performed using the Wilcoxon test. At 10 h, the *p*-value referred to NTT-FLAG and PLU-1-FLAG was 0.023, at 24 h was 0.026 and at 48 h became 0.002. At 48 h NTT-FLAG significantly differs from Empty condition with a *p*-value = 0.033. (**c**) Linear displacement of MCF7 cells under the indicated conditions, measured by live-imaging migration assay (biological triplicate). Statistical comparisons were performed using Dunn’s test: Empty vs. NTT-FLAG *p* = 0.21, Empty vs. PLU-1-FLAG *p* = 0.25, NTT-FLAG vs. PLU-1-FLAG *p* = 0.003. (**d**) Maximum distance travelled by cells in various conditions, measured by live-imaging migration assay (biological triplicate). Statistical comparisons were performed using Dunn’s test: Empty vs. NTT-FLAG *p* = 0.45, Empty vs. PLU-1-FLAG *p* = 0.52, NTT-FLAG vs. PLU-1-FLAG *p* = 0.038. (**e**,**f**) Distribution of MCF7 cells across migration classes defined by linear displacement (**e**) or maximum distance reached (**f**) in the indicated conditions. (**g**) Number of MCF7 cells counted per microscopy field after traversal of a Geltrex matrix and Transwell membrane during the invasion assay (biological triplicate). Statistical analysis was performed using the Wilcoxon test with Bonferroni correction: NTT-FLAG vs. Empty *p* = 8.08 × 10^−5^, PLU-1-FLAG vs. Empty *p* = 1.000, PLU-1-FLAG vs. NTT-FLAG *p* = 0.032. * *p* ≤ 0.05, ** *p* ≤ 0.01, *** *p* ≤ 0.001, **** *p* ≤ 0.0001.

KDM5B can act as an oncogene, favoring tumor-related phenotypes, including cellular migration and invasion. Among the biological processes identified in our previous RNA-seq dataset upon NTT-FLAG overexpression, the epithelial–mesenchymal transition (EMT) pathway was selected for further investigation, as it previously exhibited divergent, context-dependent trends across different cellular backgrounds [33]. To validate the transcriptomic alterations within this specific pathway including also a direct comparison with PLU-1-FLAG overexpression, a panel of EMT-associated DEGs was evaluated via RT-qPCR. Among the tested targets, a significant upregulation of the plasminogen activator, urokinase receptor (*PLAUR*) and colony stimulating factor 1 (*CSF-1*) genes was observed in both NTT-FLAG and PLU-1-FLAG-overexpressing cells compared with the empty-vector-treated cells (Figure 3a-*PLAUR*: Empty vs. NTT-FLAG *p*-adj = 3.02 × 10^−4^, Empty vs. PLU-1-FLAG *p*-adj = 2.6 × 10^−5^. *CSF-1*: Empty vs. NTT-FLAG *p*-adj = 4.18 × 10^−6^, Empty vs. PLU-1-FLAG *p*-adj = 1.03 × 10^−5^). Although protein-level validations are required to establish a direct causal link, these transcriptional changes suggested an activation of the EMT program, following both NTT-FLAG and PLU-1-Flag overexpression. Consequently, the migratory capability of cells under all previously described transfection conditions was tested using a wound-healing assay and live-cell imaging microscopy.

The NTT-FLAG overexpressing cells showed a faster reduction in wound area than the PLU-1-FLAG overexpressing cells after 10, 24, 48 h, and at 48 h, compared to the Empty condition (Figure 3b-NTT-FLAG vs. PLU-1-FLAG: *p* = 0.023 at 10 h, *p* = 0.026 at 24 h, and *p* = 0.002 at 48 h; NTT-FLAG vs. Empty at 48 h: *p* = 0.033). Despite that, at 48 h, the cells did not completely close the wound area, as they are very slow to migrate (Figure S4a).

Then, the cell movement was observed for 24 h by live-imaging microscopy, considering several parameters that differentiated the ability of cells to move around their initial position or in a directional way: the total distance travelled refers to the total length of the trajectory of each cell, from the initial to the final point, considering all the directional changes; the linear distance is the gap between the start and the end of the trajectory, measured as a line; the maximum (max) distance reached indicates the length in a straight-line from the starting point and the farthest point reached to the cell during its trajectory. Any differences among the Empty, NTT, and PLU-1 conditions was observed in total distance travelled (Figure S4b), whereas NTT-FLAG overexpressing cells showed higher linear and maximum distances than PLU-1-FLAG overexpressing cells, indicating greater directionality in cell movement (Figure 3c,d-Linear distance: NTT-FLAG vs. PLU-1-FLAG *p* = 0.003; Max distance: NTT-FLAG vs. PLU-1-FLAG *p* = 0.038). This observation was also supported by the per-class analysis (Figure 3e,f and Figure S4c). Indeed, the selected parameters for distances relative to the reference E-treated cells were used to define 4 rank-based distance classes: minimal, small, moderate, and large. Interestingly, the NTT-overexpressing cells showed a crescent trend in cell percentage across classes, from the minimal to the largest, with the highest percentage in the largest-distance class. In contrast, the PLU-1-overexpressing cells showed the opposite trend. This is evident, especially when considering linear distance, but also in the total and maximum distances travelled (Figure 3e,f and Figure S4c).

Finally, the invasion potential was assessed using a transwell assay with Geltrex as the extracellular matrix. As a chemotactic stimulus, a FBS gradient from 0% to 20% was obtained. After 24 h, a significantly higher number of NTT-FLAG-overexpressing cells passed the transwell membrane with respect to both the PLU-1-FLAG and empty conditions (Figure 3g and Figure S4d-NTT-FLAG vs. Empty *p* = 8.08 × 10^−5^, PLU-1-FLAG vs. NTT-FLAG *p* = 0.032).

## 3. Discussion

The current literature identifies KDMs as crucial epigenetic players in cancer-related processes. In particular, recent evidence demonstrates that the roles of KDMs are strongly context-dependent and not exclusively linked to their catalytic activity [31]. Indeed, in class 5, the development of a fully viable mouse model producing a catalytically inactive isoform and the characterization of the truncated NTT isoform in human breast cancer cells highlighted additional roles beyond the canonical one [32,33]. In particular, NTT lacks the entire JmjN domain and part of the ARID and contains an extra exon 6 that is not present in the canonical PLU-1. Although NTT is poorly transcribed, it is much more stable than the PLU-1 isoform and therefore it can accumulate in cells. In MCF7 breast cancer cells, when NTT was ectopically overexpressed, H3K4me3 in bulk chromatin increased, as proved by mass spectrometry, and several genes were transcriptionally modulated, as previously described [33]. These observations suggest that NTT may act as a negative competitor of the catalytically functional PLU-1. Thus, according to our hypothesis, NTT may retain the ability to interact with chromatin via its PHDs, but being unable to demethylate, it may protect binding sites from the action of other demethylases (PLU-1, KDM5A, or KDM5C).

**Figure 4:**
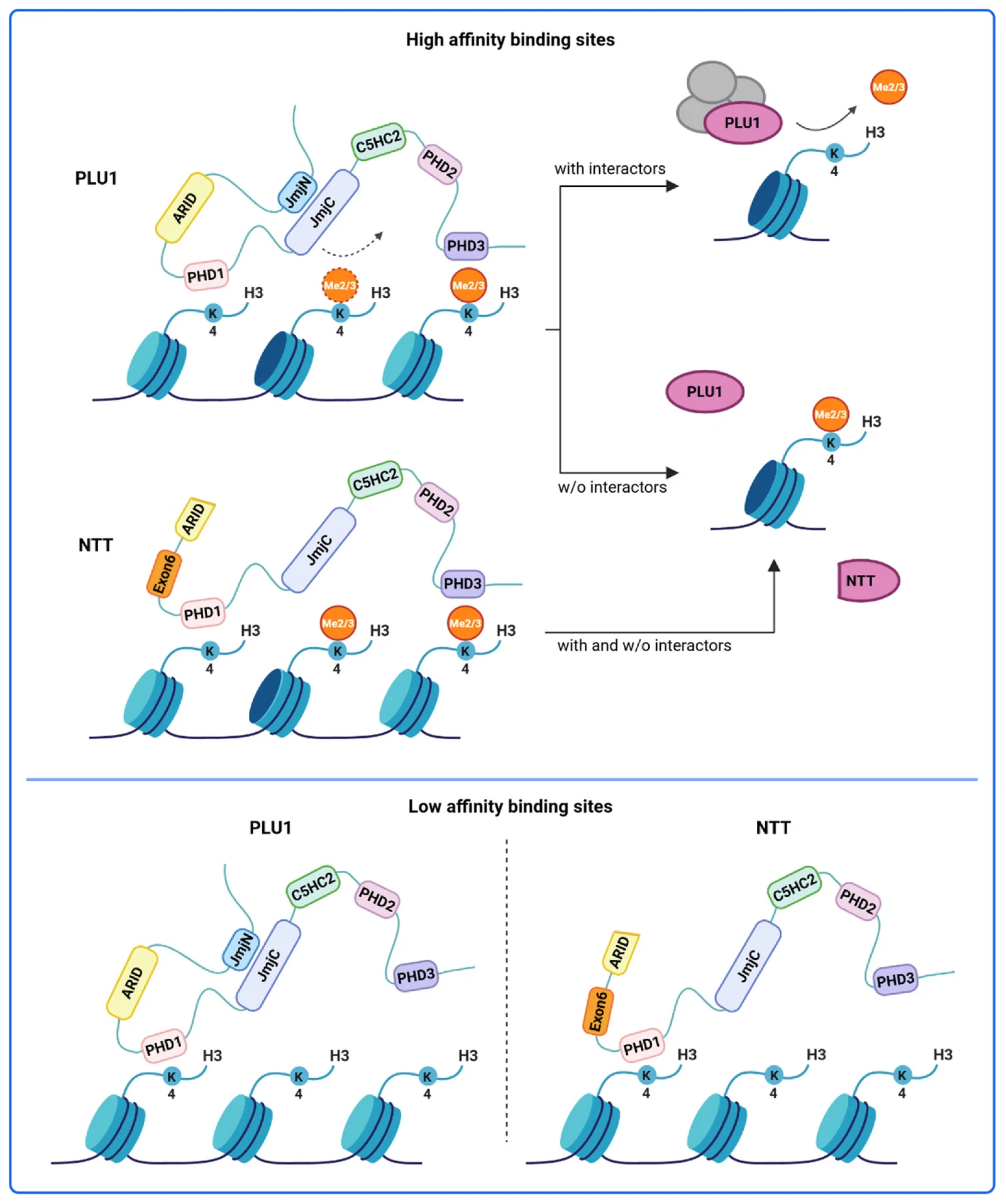
Proposed model for chromatin binding and H3K4me3 regulation by KDM5B isoforms. The model integrates the distinct functional properties of NTT and PLU-1 to explain isoform-specific outcomes at chromatin regulatory elements. Both KDM5B isoforms are proposed to scan chromatin via transient, low-affinity interactions mediated by the PHD1 domain, which recognises unmethylated H3K4 (H3K4me0). These transient contacts are here referred to as low-affinity binding sites. When such interactions occur in proximity to H3K4me3-containing regions, the PHD3 domain may additionally be engaged to interact with the trimethylated mark, stabilizing the interaction into a high-affinity binding site. Here, PLU-1, which retains its catalytic potential, could demethylate H3K4me3; however, full demethylase activity is proposed to require the cooperative action of a set of interactors. They are hypothesized to be present in limiting amounts upon ectopic overexpression of PLU-1, and are presumed to be preferentially engaged by the highly abundant endogenous isoform in MCF7 cells. Under these conditions, overexpressed PLU-1 may occupy high-affinity sites without effectively demethylating H3K4me3. On the contrary, NTT, being catalytically inactive, would never demethylate H3K4me3 regardless of cofactor availability. Nevertheless, its occupancy at high-affinity sites could competitively shield these regions from demethylation by other KDMs, including endogenous PLU-1. The dashed arrow indicates the possible removal of the involved epigenetic target (dashed methyl group) by catalytic activity. The solid arrow indicates the demethylation. Figure created with BioRender.com.

This effect was mostly evident when NTT-FLAG was overexpressed in MCF7 cells where the ratio between the endogenous PLU-1 and NTT isoforms is quite high, making this cell line a suitable model to prove this concept. For these reasons, in this work, the MCF7 cell line was selected. Indeed, the hypothesis was supported by CUT&RUN analysis. This study showed that, despite the lack of part of the DNA-interacting ARID domain, ectopically expressed NTT efficiently associated with genomic chromatin in MCF7 cells, likely via its PHDs, thereby recapitulating the PLU-1 mode of interaction. Both isoforms tend to associate with efficiently transcribed genes enriched for H3K4me3 peaks on promoters and probably characterized by an open chromatin state. FLAG-tagged NTT and PLU-1 often localize around the TSS and within promoters, as previously reported for canonical PLU-1 [17]. Moreover, both of them show multiple binding sites within gene bodies, introns and distal intergenic regions as previously observed [17,25]. However, the FLAG peaks referred to both NTT and PLU-1 binding show low exact coincidence across replicates, highlighting high dynamics of chromatin association. Indeed, KDM5B transiently interacts with chromatin at multiple sites, functioning either as a reader or an eraser. Conversely, strong reproducibility of H3K4me3 signals across replicates and conditions was observed, as it is a highly stable histone modification. All these observations are consistent with the involvement of PHD1 domains, which, by recognizing H3K4me0, interact with chromatin and spread across the entire genome. NTT and PLU-1 may use a system like this to explore the genome in a low-affinity, highly transient manner while maintaining contact with chromatin. The presence of H3K4me3 in many targets suggests a preference for NTT and PLU-1 to interact with trimethylated regions. Probably, as shown in Figure 4, when H3K4me3 is present, it recruits the proteins through their PHD3 domain, which stabilizes chromatin interactions in these regions, as supported by Klein et al., who identified PHD3 as the major driver of KDM5B-chromatin interaction [25].

Next, the effects of the NTT-FLAG and PLU-1-FLAG association on H3K4 tri-methylation at specific genomic sites were examined. While an increase in H3K4me3 was expected in NTT-FLAG-overexpressing cells, due to the putative dominant-negative role of the inactive protein, a similar effect in PLU-1-FLAG-overexpressing cells was surprising. One possible explanation is that the FLAG-tagged PLU-1 was not entirely functional, even though it produced a fully functional protein in vitro [34]. Another hypothesis is that, as previously proposed [17], the association with chromatin is not sufficient to activate demethylation and corepressor activity (CHD4, HDAC1, CTCF, others) is required to stabilize PLU-1 association and/or to support its catalytic activity, for example, by introducing post-translational modifications necessary to trigger efficient catalytic activity (Figure 4). Indeed, a recent study showed that a KDM5 variant outside the catalytic Jumonji C domain which alters the proximity interactome disrupts demethylase activity, suggesting that proper complex assembly may be a mechanistic prerequisite for catalysis [35]. Presumably, the interacting corepressors are already engaged by the endogenous KDM5B, which is very abundant in MCF7 cells. To verify this interpretation, H3K4me3 levels were measured by Western blot analysis in HEK293, an embryonic kidney cell line that has a lower basal KDM5B expression than MCF7, after PLU-1-FLAG and NTT-FLAG overexpression and could provide free corepressors to the overexpressed demethylase. Indeed, we observed a significant decrease in the level of H3K4me3 in the bulk chromatin of the HEK293 cells transfected with PLU-1-FLAG compared with those transfected with NTT-Flag. This result strongly suggests the functionality of PLU-1-FLAG demethylase activity in those cells.

The availability of corepressors for ectopically expressed PLU-1-FLAG would, of course, depend on its basal expression in different cells. This would explain why the transcriptomic effects of ectopically expressed KDM5B are highly variable across cell lines and limited in those that show very high basal levels of the endogenous protein, such as MCF7 [27]. Notably, NTT-FLAG was associated with 32 of the 123 deregulated genes after NTT-FLAG overexpression, as previously identified by RNA-seq analysis [33]. For some of them, a possible involvement in transcriptional regulation was suggested, as H3K4me3 occupancy increased after isoform overexpression. For the other genes, alternative, still-unknown mechanisms or indirect effects should be considered. As an example, *CSF-1*, which can be induced by ectopic overexpression of NTT-FLAG, does not show increased H3K4me3 at its promoter. Consistent with these observations, an independent study affirmed that alterations in H3K4me3 levels may not be sufficient to induce transcriptional changes and that interactions with multiple regulatory factors or complexes may be required [35]. Indeed, PLU-1, and likely NTT as well, interact with several proteins (i.e., transcription factors) that could be involved in transcriptional effects. Moreover, E-promoters, which combine promoter and enhancer features, represent highly complex regulatory hubs where an extensive network of factors and chromatin remodelers are recruited [36]. Thus, structural differences among KDM5B isoforms may perturb this intricate network of interactions, providing an additional layer of transcriptional regulation.

KDM5B has been widely described as an oncogene that promotes several tumor-associated phenotypes, including increased cellular migration and invasion [37,38]. Consistent with this role, the previous RNA-seq analysis in MCF7 cells revealed that NTT-FLAG overexpression induces biological pathways related to epithelial–mesenchymal transition (EMT) [33], a key process involved in tumor progression and metastatic dissemination. The EMT pathway was specifically selected for further investigation because it previously exhibited context-dependent, divergent trends across different cellular backgrounds. A panel of EMT-associated transcripts was evaluated via RT-qPCR to screen for target modulation shared between NTT and PLU-1 conditions, leading to the identification of *CSF-1* and *PLAUR* upregulation in both NTT-FLAG and PLU-1-FLAG-overexpressing cells compared with empty-vector-treated cells. While these two genes are extensively documented in the literature for their potential to enhance the invasive capacity of tumor cells [39,40,41,42,43,44,45,46], these findings must be considered purely descriptive at this stage. Indeed, since protein-level validations are required to establish a direct causal link between such transcriptional changes and the functional phenotype, these variations simply suggest an activation of the EMT program, which was further investigated through phenotypic assays.

The migration assays, used to study how these transcriptional changes translated into function, revealed that NTT-FLAG overexpression promotes a more pronounced migratory phenotype than PLU-1-FLAG and control conditions. In particular, live-cell microscopy revealed that NTT-FLAG-overexpressing cells exhibited more directed migration than PLU-1-FLAG-overexpressing cells. In the two overexpression conditions, the total distance travelled did not change, but NTT-FLAG cells appeared to migrate greater linear distances. This suggests that NTT may act on the directionality of migration rather than inducing a higher migration capability. In line with these observations, the invasion assay also showed increased invasiveness in the NTT-FLAG condition, with a significantly higher number of NTT-FLAG overexpressing cells invading through the membrane compared with both PLU-1-FLAG overexpressing and empty-vector control cells. This result further supports the hypothesis that NTT-FLAG overexpression may enhance tumor-related phenotypes associated with cell invasion.

Overall, our findings support the hypothesis that the truncated KDM5B isoform NTT can interact with chromatin despite lacking catalytic activity and the ARID domain. At least in conditions of overexpression, it may influence H3K4me3 states and transcription by interfering with the function of the canonical PLU-1 isoform or by indirectly recruiting on-site-specific co-interactors. It remains to be verified how this is relevant in physiological conditions, depending on the PLU-1/NTT ratio. Through these mechanisms, NTT may contribute to transcriptional and phenotypic changes associated with tumor progression. This work highlights that the non-catalytic functions of KDM5B operate independently of its primary demethylase role, demonstrating that these non-canonical roles are at least as important as the catalytic ones in cellular regulation. This study presents some limitations, as the molecular and functional characterizations were performed using ectopic expression with FLAG-tagged constructs. Moreover, the low number of biological replicates for CUT&RUN limits the robustness of peak overlap analysis. Thus, these findings need to be validated by increasing the number of biological replicates and under physiological conditions. The current work was restricted to a single cellular model, so expanding these analyses to a broader panel of cancer cell lines will be necessary. Furthermore, proteomic analyses would precisely determine the involvement of specific DEGs containing NTT-binding sites in cancer-related phenotypes.

## 4. Materials and Methods

### 4.1. Cell Cultures

The MCF7 (luminal-like, ER-positive) human breast cancer cell line was primarily used, while the embryonic kidney HEK293 cell line was selected to test PLU-1-FLAG demethylase activity. Cells were cultured under adherent conditions at 37 °C, 5% CO_2_, in high-glucose DMEM (4.5 g/L; Corning, #15-013-CV, Corning, NY, USA) supplemented with 10% FBS (S.I.A.L. srl, Rome, Italy), 1% L-glutamine (1 mM; Gibco, Grand Island, NY, USA), and 1% penicillin/streptomycin (100 U/mL and 100 µg/mL; Gibco, Grand Island, NY, USA). Cells were stored in liquid nitrogen until use. For passaging, cells were detached using trypsin/EDTA (Corning, #25-053-CI, Corning, NY, USA) and split at 1:4–1:8 ratios. Mycoplasma contamination was periodically tested using the Mycoplasma PCR Detection Kit (Abcam, #ab289834, Cambridge, UK).

### 4.2. Cellular Transfection

Four plasmid vectors were used for transfection: empty control (pcDNA3.1(+)-C-DYK; E), NTT-FLAG overexpression vector (KDM5B_OHu64359D_pcDNA3.1+ C-(K)-DYK; NTT), PLU1-FLAG overexpression vector (KDM5B_OHu27995D_pcDNA3.1+ C-(K)-DYK; PLU1), and GFP expression vector (pcDNA3.1(+)-C-eGFP; GFP) (Figure S1). GenScript (Piscataway, NJ, USA) synthesised all plasmids. Plasmids were amplified in Escherichia coli DH5α and purified using the QIAfilter Plasmid Midi Kit (Qiagen, #12243, Venlo, The Netherlands). Plasmid integrity was verified by restriction enzyme digestion followed by agarose gel electrophoresis.

Cell transfections were performed using Lipofectamine 3000 (Invitrogen, Thermo Fisher Scientific, #L3000001, Carlsbad, CA, USA). Cells were seeded at 30,000 cells/cm^2^ and incubated at 37 °C, 5% CO_2_. After 24 h, the medium was replaced with antibiotic-free medium and transfection was carried out following the manufacturer’s instructions for the MCF7 cell line, optimising the protocol to use 4 µg of plasmid DNA per 35 mm well.

For H3K4me3 detection experiments, MCF7 and HEK293 were transfected using PEI reagent (Sigma-Aldrich, #765090, St. Louis, MO, USA) at a 1:3 DNA: PEI ratio. DNA amounts were defined according to the Lipofectamine 3000 optimised protocol.

Experimental conditions included: untreated control (CT), empty vector (E), NTT overexpression (NTT), and PLU1 overexpression (PLU1). A GFP vector was used to assess transfection efficiency. All reagents were diluted in Opti-MEM (Gibco, #31985062, Grand Island, NY, USA). Next, changes in the presence of H3K4me3 at genes and promoters. 

### 4.3. RNA-Seq

The RNA-seq experimental method was previously described in detail [33]. In this study, the obtained data were analyzed using the Join tool on the Galaxy platform (https://usegalaxy.org/, accessed on 29 July 2025) for peak merging. Statistical analyses were generated using R (v4.5) and RStudio (v2026.01).

### 4.4. Cleavage Under Targets and Release Using Nuclease (CUT&RUN) Assay

CUT&RUN was performed on MCF7 cells seeded at 30,000 cells/cm^2^ in 60 mm dishes. Cells were transfected after 24 h with E, NTT-FLAG, or PLU1-FLAG plasmids and collected 24 h post-transfection. Approximately 100,000 cells per condition were used for CUT&RUN with the anti-H3K4me3 or anti-FLAG antibodies. Each condition was analyzed in two independent biological replicates, plus IgG control. The assay was performed using the CUT&RUN Assay Kit (Cell Signalling, #86652, Danvers, MA, USA), with the anti-H3K4me3 antibody (Cell Signalling Technology, #9751, Danvers, MA, USA), the anti-FLAG M2 antibody (Sigma, #F1804, St. Louis, MO, USA), and the Rabbit IgG isotype control (Cell Signalling Technology, #66362, Danvers, MA, USA).

FASTQ files were quality-checked using FastQC (v0.12.1) and trimmed with Trim Galore! (v0.6.10). Initial raw sequencing depths ranged from 6.06 to 22.35 million reads per sample, which, after the removal of adapter sequences and low-quality bases, yielded between 5.9 and 21.82 million clean reads per sample. The overall sequencing quality metrics confirmed robust library preparation, characterized by a stable base error rate of 0.01%, with Q30 percentages between 97.30% and 97.46%. Following read filtering, the clean reads were aligned to the reference genome and the total mapping rate spanned from 60.0% to 92.9% with uniquely mapped reads accounting for 50.7% to 85.0%. Ultimately, all samples are above standard bioinformatic thresholds for CUT&RUN quality. Reads were aligned to the reference genome using Bowtie2 (v2.5.3), then sorted and indexed with SAMtools (v1.20), and duplicates marked with Picard (v3.1.1). Coverage files and signal tracks were generated using deepTools (v3.5.5) and bedGraphToBigWig (v. 445). BAM files were normalized by library size using Counts Per Million (CPM) normalization to generate bigWig files and any resulting negative signal values were set to zero. Peak calling (Table S3) was performed with SEACR (v1.4), and genomic interval operations were conducted using BEDTools (v2.31.1). We used “Stringent mode” SEACR output to focus exclusively on the most robust binding regions and eliminate as much as possible noise-level signals and use the peak of the total signal curve to maximize specificity and minimize false positives automatically. SEACR does not calculate a traditional False Discovery Rate (FDR) or use statistical models. Instead, it uses an empirical, data-driven approach based on the global distribution of background (IgG control) signal [47]. Quality metrics were summarised with MultiQC (v1.22). Peak merging was performed using the Join tool on Galaxy platform (https://usegalaxy.org/, accessed on 29 July 2025). Statistical analyses and plots were generated using R (v4.5) and RStudio (v2026.01).

Anti-FLAG peaks identified in each replicate of the NTT-FLAG and PLU-1-FLAG overexpression conditions were compared with those detected in the corresponding empty control replicate based on their genomic coordinates using the Galaxy platform. Individual peaks showing an overlapping of at least 1bp with those detected in the respective empty condition were removed from each overexpression dataset to exclude potentially non-specific anti-FLAG enrichment.

Gene expression for NTT-FLAG overexpression from NBCI’s Gene Expression Omnibus (GEO) repository accession number GSE218819 was analyzed as previously described and data were normalized using the R/Bioconductor DESeq2 package (v1.50). DESeq2 normalizes RNA-seq data using the median-of-ratios method, dividing each sample’s raw counts by a sample-specific size factor calculated from the median ratio of a gene’s count to its geometric mean across all samples to correct for differences in sequencing depth and library composition.

### 4.5. Protein Extraction and Quantification

Proteins were extracted 24 h post-transfection using RIPA buffer (Sigma-Aldrich, #R0278, St. Louis, MO, USA) supplemented with protease (Roche, #11697498001, Basel, Switzerland) and phosphatase inhibitors (Sigma, #4906845001, St. Louis, MO, USA). Cells were washed twice with DPBS (S.I.A.L. srl, #SIAL-PBS-1A, Rome, Italy), lysed in RIPA buffer, and incubated on ice for 15 min. Lysates were clarified by centrifugation at 13,000× *g* for 25–30 min at 4 °C, and supernatants were collected and stored at −80 °C.

For H3 and H3K4me3 analysis, nuclear proteins were extracted as previously described [48]. Briefly, cells were transfected with PEI and collected 24 h later. Pellets were washed with PBS and resuspended in Nuclei Isolation Buffer (PBS, 0.1% Triton X-100, protease inhibitors), followed by centrifugation at 2300× *g* for 15 min at 4 °C to isolate nuclei. Nuclear pellets were resuspended in NIB containing 0.1% SDS and benzonase and incubated at 37 °C with shaking. Nuclear extracts were clarified by centrifugation at 13,000× *g* for 30 min at 4 °C and stored at −80 °C.

Protein concentration was measured using the Pierce Bradford Protein Assay Kit (Thermo Fisher Scientific, #23200, Carlsbad, CA, USA) according to the manufacturer’s instructions. Samples and standards were measured in duplicate.

### 4.6. Western Blot Analysis

SDS-PAGE Western blot was performed to detect KDM5B isoforms, H3, and H3K4me3. Total protein lysates (25–30 µg) were mixed with 4× Laemmli buffer (Bio-Rad, #161-0747, Hercules, CA, USA) containing 10% β-mercaptoethanol, denatured at 70 °C for 10 min, and loaded into polyacrylamide gels. KDM5B isoforms were resolved on 5% gels (37.5:1 acrylamide/bisacrylamide, Sigma-Aldrich, #A3699, St. Louis, MO, USA), whereas H3 and H3K4me3 were separated on 12% gels (29:1 acrylamide/bisacrylamide, Sigma-Aldrich, #A2792, St. Louis, MO, USA). Electrophoresis was performed in TGS buffer (Bio-Rad, #161-0732, Hercules, CA, USA) using a pre-stained protein ladder (Thermo Fisher Scientific, #26625, Carlsbad, CA, USA). Proteins were transferred onto nitrocellulose membranes (Amersham, #GE10600007, Buckinghamshire, UK) in TG buffer (Bio-Rad, #161-0734, Hercules, CA, USA) at 100 V for 2 h (5% gels) or 90 V for 90 min (12% gels). Membranes were stained with Ponceau S to verify equal loading, then washed in TBS-T (0.1% Tween-20), blocked in 5% milk for 1 h, and incubated overnight at 4 °C with the following primary antibodies: anti-KDM5B (Atlas Antibodies, #HPA0217179, Bromma, Sweden; 1:7000 in 5% BSA), anti-H3 (Abcam, #ab1791, Cambridge, UK; 1:1000 in 5% milk), and anti-H3K4me3 (Abcam, #ab8580, Cambridge, UK; 1:1000 in 5% BSA). After washing, membranes were incubated with HRP-conjugated goat anti-rabbit secondary antibody (Thermo Fisher Scientific, #31460, Carlsbad, CA, USA) for 1 h at room temperature. Signal was detected using the Clarity ECL substrate (Bio-Rad, Hercules, CA, USA) and imaged with the ChemiDoc MP (Bio-Rad, Hercules, CA, USA). Western blot images were analyzed using Image Lab version 6.1 software.

### 4.7. Wound Healing Assay

Cell migration was assessed using Culture-Insert 2 Well in µ-Dish 35 mm (IBIDI, #81176, Gräfelfing, Germany). Cells were seeded at 30,000 cells/cm^2^ and transfected directly within the inserts 24 h later. Insert removal the following day, defined time 0 h. Cells were maintained in serum-free medium to minimise proliferation.

For each wound, two images were acquired at distinct locations at 0, 10, 24, and 48 h using the ZOE Fluorescence Cell Imager (Bio-Rad, Hercules, CA, USA). Wound area was quantified using the ImageJ (1.53e/Java 1.8.0_172) plugin Wound Healing Size, and data were normalized to the respective 0 h time point. Results are presented as mean ± SD. Statistical significance was assessed using Student’s *t*-test and the Wilcoxon test. Graphs and analyses were generated using RStudio (2024.04.0).

### 4.8. Live-Imaging Microscopy

For experiments in live-imaging microscopy, MCF7 cells were first seeded into 35 mm dishes (30,000 cells/cm^2^) and transfected 6–8 h later. After 16–18 h, cells were detached, counted using a Bürker chamber, and reseeded in µ-Slide 8 Well high chambers (IBIDI, #80806, Gräfelfing, Germany) at 3500 cells/well. Following 4–6 h for attachment, slides were transferred to a microscope incubator (Okolab Basic WJ, Pozzuoli, Italy), and images were acquired every 30 min for 24 h using a Nikon Ti (Tokyo, Japan) inverted microscope equipped with a Clara camera (ANDOR, Belfast, UK; 10× objective). Three independent biological replicates were performed, each including four different microscopy fields per condition as technical replicates.

Time-lapse sequences were analyzed using the TrackMate plugin in FIJI (ImageJ 2.140/1.49v/Java 1.8.0_322). Cell detection was performed using a defined diameter of 35 µm, and trajectories were reconstructed across frames. Tracks were filtered based on: number of spots (≥15), number of gaps (≤2), longest gap (≤5 frames), and track duration (two-thirds of total frames). Extracted parameters included track displacement (µm), total distance travelled (µm), and maximum distance travelled (µm). Only tracks with displacement ≥ detection diameter were retained to exclude non-motile cells. Time-lapse experiments have been performed at the IBPM-Nikon Reference Center.

Statistical analyses and graphical representations were performed using RStudio (2024.04.0). The Wilcoxon test was used for boxplot comparisons; Fisher’s test was applied for categorical analyses.

### 4.9. RNA Purification and RT-qPCR

Total RNA was extracted 24 h post-transfection using the RNeasy Mini Kit (Qiagen, #74104, Venlo, The Netherlands) according to the manufacturer’s instructions. RNA concentration and purity were assessed using a NanoDrop One spectrophotometer (Thermo Fisher Scientific, Carlsbad, CA, USA). RNA was stored at −80 °C.

1 µg of RNA was reverse-transcribed using the SensiFAST cDNA Synthesis Kit (Meridian Biosciences, #BIO-65053, Cincinnati, OH, USA) following the manufacturer’s protocol. cDNA was diluted 1:3 with DNase/RNase-free water before qPCR.

Primers were designed using Primer-BLAST (NCBI—https://www.ncbi.nlm.nih.gov/tools/primer-blast/, accessed on 29 July 2025) according to standard parameters (length 20–23 nt, GC content ~50%, Tm 55–65 °C) and synthesised by Biofab Research (Rome, Italy). Primer efficiency was validated by standard and melting-curve analyses; only primer pairs with optimal slopes (~–3.32) and R^2^ values were used (Table S2).

qPCR was performed using the SensiFAST SYBR Hi-ROX Kit (Meridian Bioscience, #BIO-92005, Cincinnati, OH, USA) on a StepOnePlus Real-Time PCR System (Thermo Fisher Scientific, Carlsbad, CA, USA). Reactions (10 µL) contained 2× SYBR mix, primers (0.2 µM final concentration), and diluted cDNA. Cycling conditions were: 95 °C for 2 min, followed by 40 cycles of 95 °C for 5 s and 60 °C for 30 s.

Relative gene expression was calculated using the 2^−ΔΔCt^ method, with β-actin as the endogenous control and the empty vector condition as reference. Five independent biological replicates were analyzed. Statistical significance was assessed using Student’s *t*-test, and analyses were performed in RStudio (2024.04.0). Next, changes in the presence of H3K4me3 at genes and promoters.

### 4.10. Trans-Well Invasion Assay

For the invasion assay, MCF7 cells were first seeded and transfected in 6-well plates as previously described, then incubated at 37 °C and 5% CO_2_ overnight. In the meantime, the transwell chambers were coated by diluting Geltrex (Gibco, #A1413301, Grand Island, NY, USA) 1:60 in DMEM *w*/*o* FBS, adding 100 µL to each transwell, and incubating overnight at 37 °C. The next day, the cells were detached and counted, and the coating medium was discarded and replaced with 200 µL of DMEM containing 1% L-glut, 0% FBS and 1500 cells per well per condition. Transwell chambers were settled in 24-well plates, and the bottom compartment was filled with 750 µL of DMEM with 1% L-glut and 20% FBS. After 24 h, when cells were free to invade the Geltrex and the Transwell membrane, cells were fixed and stained. In particular, the medium was discarded from both the top and bottom of the chambers, the chambers were washed twice with PBS, and then maintained in a solution of 4% Formaldehyde in PBS for 20 min. Again, the chambers were washed twice with PBS and stained for 20 min in a solution of 0.5% Crystal Violet and 2% EtOH in distilled water. After the final washes, images were captured using the AE2000 Inverted Microscope (Motic, Xiamen, China) in 6 different microscopic fields per condition. Three biological replicates were performed. Statistical analyses and graphs were generated using RStudio (2024.04.0). *p*-values were calculated by Student’s *t*-test with Bonferroni correction.

## 5. Conclusions

This study provides further insight into the non-catalytic functions of KDM5B by characterizing the behavior of its truncated isoform, NTT. Our initial hypothesis proposed that NTT could act as a dominant competitor of the canonical PLU-1 isoform, retaining the ability to interact with chromatin at similar target sites without performing demethylase activity. The current results indicate that NTT maintains the ability to associate with chromatin, likely through its PHD domains, preferentially targeting actively transcribed regions enriched in H3K4me3. Despite lacking demethylase activity, NTT apparently influences chromatin states by promoting a modest H3K4me3 accumulation at specific genomic loci and modulating gene-associated processes, which a similar behavior observed in PLU-1-FLAG overexpressing cells. The proposed model suggests that NTT and PLU-1 may dynamically scan chromatin in a transient and low-affinity manner via PHD1, while H3K4me3 may stabilize their interaction by recruiting PHD3. Importantly, chromatin binding alone may not be sufficient to drive enzymatic or repressive functions, which likely require the recruitment of corepressor complexes. The availability of these cofactors may depend on the endogenous levels of KDM5B, which likely preferentially associates with them. Overall, these findings underscore the importance of considering both enzymatic and non-enzymatic roles of KDM5B isoforms in cancer biology, providing a descriptive framework for further investigation into their mechanistic and therapeutic relevance.

## Figures and Tables

**Table 1:** Differentially Expressed Genes (DEGs) identified by RNA-seq (NTT-FLAG vs. empty-vector-treated cells) harboring binding sites for NTT-FLAG and PLU-1-FLAG. The table summarizes a subgroup of DEGs selected for the presence of NTT-FLAG or PLU-1-FLAG peaks within their length. The last two columns report, for each isoform, the genomic localization of the peak and the corresponding number of binding peaks. Genes bound by both isoforms display values in both columns, whereas genes bound by only one isoform display values exclusively in the corresponding column.

Symbol	Name	log2FoldChange *	NTT-FLAG Peaks (*n*)	PLU-1-FLAG Peaks (*n*)
*TFCP2L1*	Transcription Factor CP2 Like 1	4.635	Distal Intergenic (2), Intron (2), 5′ UTR (1)	Distal Intergenic (4)
*AZGP-1*	Alpha-2-Glycoprotein 1, Zinc-Binding	4.565	3′ UTR (1), Intron (1)	Intron (1)
*SAMD-9*	Sterile Alpha Motif Domain Containing 9	3.943	Distal Intergenic (17), Intron (8), Exon (1)	Intron (5), Distal Intergenic (11)
*OAS-1*	2′-5′-Oligoadenylate Synthetase 1	3.576	Promoter (1), Distal Intergenic (1)	Exon (1)
*NAV-2*	Neuron Navigator 2	3.536	Distal Intergenic (3), Intron (2)	Exon (1), Intron (1)
*IFIT-3*	Interferon Induced Protein With Tetratricopeptide Repeats 3	3.314	Distal Intergenic (1), Intron (1)	Distal Intergenic (4), 3′ UTR (1)
*SLC16A7*	Solute Carrier Family 16 Member 7	3.312	Promoter (2), Intron (5), Distal Intergenic (1)	Promoter (1), Intron (3), Distal Intergenic (2)
*OASL*	2′-5′-Oligoadenylate Synthetase Like	3.249	Intron (1)	3′ UTR (1)
*MX-2*	MX Dynamin Like GTPase 2	3.026	Promoter (2), Exon (1), Intron (2)	Promoter (2)
*CAV-1*	Caveolin 1	2.224	Promoter (1), 3′ UTR (1)	Promoter (1)
*CSF-1*	Colony Stimulating Factor 1	1.688	Promoter (1), Intron (4)	Exon (2), Intron (1)
*TAGLN*	Transgelin	1.688	Intron (2)	Promoter (1)
*TAP-2*	Transporter 2, ATP Binding Cassette Subfamily B Member	0.939	3′ UTR (1), Exon (1)	3′ UTR (2)
*AFF-1*	ALF Transcription Elongation Factor 1	0.734	Intron (1)	Exon (1)
*UGDH*	UDP-Glucose 6-Dehydrogenase	0.587	Promoter (1), Intron (1)	Distal Intergenic (1), Intron (2)
*SKAP-2*	Src Kinase Associated Phosphoprotein 2	0.555	Promoter (1), Distal Intergenic (1)	Intron (5)
*CNP*	2′,3′-Cyclic Nucleotide 3′ Phosphodiesterase	0.503	Exon (1)	Exon (1)
*TAPBP*	TAP Binding Protein	0.460	Promoter (1)	Promoter (1)
*AGRN*	Agrin	0.270	Promoter (1)	Exon (1)
*CCSER-2*	Coiled-Coil Serine Rich Protein 2	−0.595	Distal Intergenic (1), Intron (1)	Promoter (1), Intron (2)
*C5*	Complement C5	−0.724	Promoter (1), Exon (2), Intron (5)	Exon (3), Intron (2)
*TF*	Transferrin	−3.516	Intron (1)	Promoter (1), Intron (3)
*CXCL-10*	C-X-C Motif Chemokine Ligand 10	5.527	Intron (1)	-
*RSAD-2*	Radical S-Adenosyl Methionine Domain Containing 2	5.425	Intron (1)	-
*OAS-2*	2′-5′-Oligoadenylate Synthetase 2	5.231	Exon (2)	-
*IL-32*	Interleukin 32	4.361	Promoter (1)	-
*DDX-60*	DExD/H-Box Helicase 60	3.827	Intron (1)	-
*IFIT-1*	Interferon Induced Protein With Tetratricopeptide Repeats 1	3.655	Intron (1)	-
*ICAM-1*	Intercellular Adhesion Molecule 1	1.180	Exon (1)	-
*HLA-B*	Major Histocompatibility Complex, Class I, B	1.024	Promoter (1)	-
*NR4A1*	Nuclear Receptor Subfamily 4 Group A Member 1	0.901	Promoter (1)	-
*VDR*	Vitamin D Receptor	0.711	Exon (2), Intron (4), 3′ UTR (1)	-
*UBA-7*	Ubiquitin Like Modifier Activating Enzyme 7	3.790	-	Promoter (1)
*CCL-5*	C-C Motif Chemokine Ligand 5	3.182	-	Exon (1)
*IRF-9*	Interferon Regulatory Factor 9	1.484	-	Exon (1)
*RELB*	RELB Proto-Oncogene, NF-KB Subunit	1.357	-	Intron (1)
*TRIM-21*	Tripartite Motif Containing 21	1.213	-	Promoter (1), Exon (1)
*CEP-250*	Centrosomal Protein 250	−0.496	-	Promoter (1)

* Referred to DEGs regulation in RNA-seq analysis. All DEGs reported are associated with FDR < 0.05.

## Data Availability

The CUT&RUN data accompanying this paper are available through NCBI’s Gene Expression Omnibus (GEO) repository, under accession number GSE341885 located at https://www.ncbi.nlm.nih.gov/geo/query/acc.cgi?acc=GSE341885 (accessed on 31 July 2026).

## Supplementary Materials

The following supporting information can be downloaded at: https://www.mdpi.com/article/10.3390/ijms27198774/s1.
