# Supplementary material for: The Catalytically Inactive KDM5B-NTT Isoform Associates with Chromatin and Increases H3K4 Trimethylation in Breast Cancer Cells
Source: Int J Mol Sci. 2026 Sep 30;27(19):8774. doi: 10.3390/ijms27198774 (PMC13664838; doi:10.3390/ijms27198774)
Supplement: Supplementary file 1 [file ijms-27-08774-s001.zip › FigureS1.pdf]

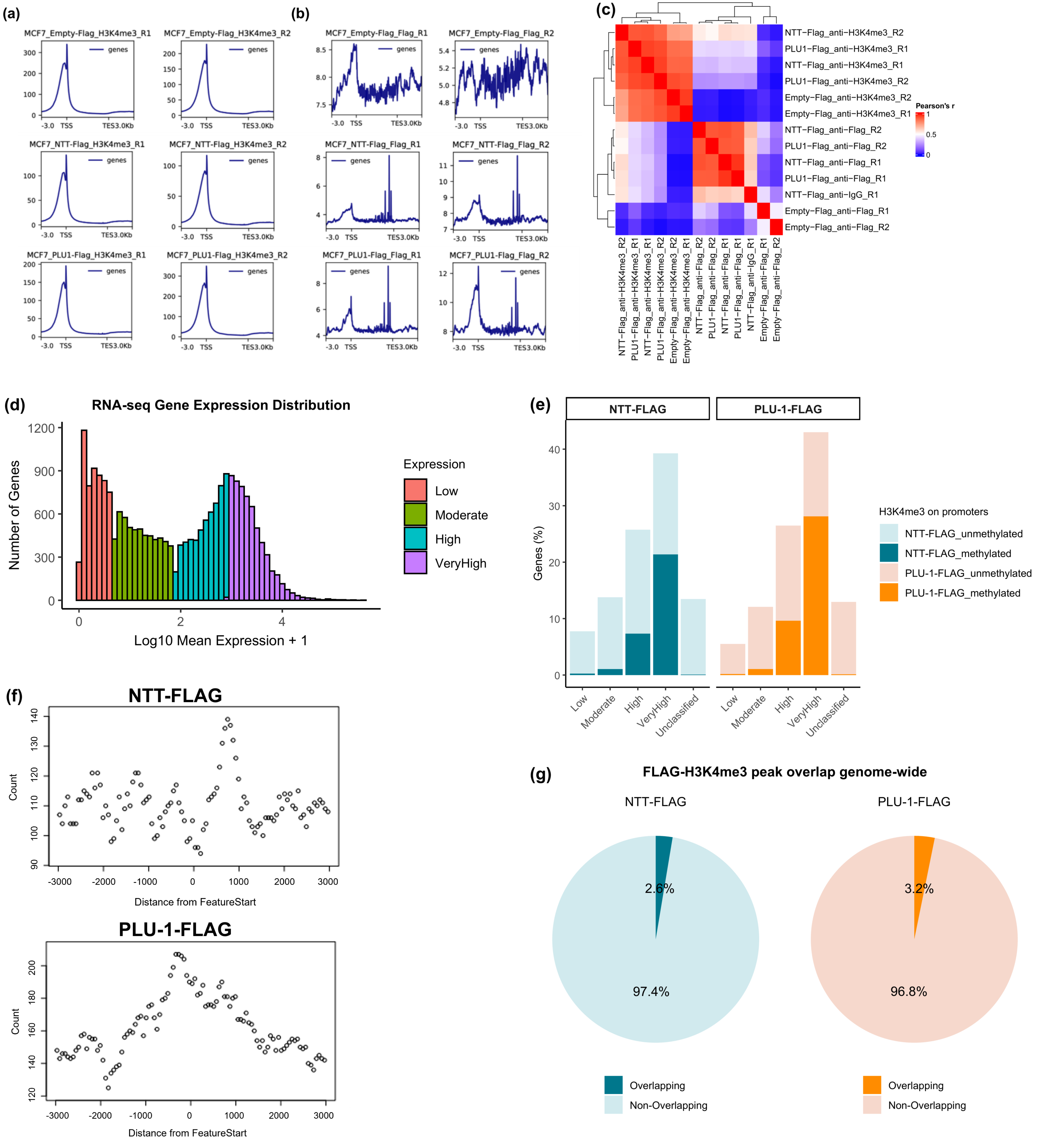

(a)
MCF7_Empty-Flag_H3K4me3_R1
MCF7_Empty-Flag_H3K4me3_R2
MCF7_NTT-Flag_H3K4me3_R1
MCF7_NTT-Flag_H3K4me3_R2
MCF7_PLU1-Flag_H3K4me3_R1
MCF7_PLU1-Flag_H3K4me3_R2
genes
-3.0 TSS TES3.0Kb
(b)
MCF7_Empty-Flag_Flag_R1
MCF7_Empty-Flag_Flag_R2
MCF7_NTT-Flag_Flag_R1
MCF7_NTT-Flag_Flag_R2
MCF7_PLU1-Flag_Flag_R1
MCF7_PLU1-Flag_Flag_R2
(c)
NTT−Flag_anti−H3K4me3_R2
PLU1−Flag_anti−H3K4me3_R1
NTT−Flag_anti−H3K4me3_R1
PLU1−Flag_anti−H3K4me3_R2
Empty−Flag_anti−H3K4me3_R2
Empty−Flag_anti−H3K4me3_R1
NTT−Flag_anti−Flag_R2
PLU1−Flag_anti−Flag_R2
NTT−Flag_anti−Flag_R1
PLU1−Flag_anti−Flag_R1
NTT−Flag_anti−IgG_R1
Empty−Flag_anti−Flag_R1
Empty−Flag_anti−Flag_R2
Pearson's r
(d)
RNA-seq Gene Expression Distribution
Number of Genes
Log10 Mean Expression + 1
Expression
Low
Moderate
High
VeryHigh
(e)
NTT-FLAG
PLU-1-FLAG
Genes (%)
Low
Moderate
High
VeryHigh
Unclassified
H3K4me3 on promoters
NTT-FLAG_unmethylated
NTT-FLAG_methylated
PLU-1-FLAG_unmethylated
PLU-1-FLAG_methylated
(f)
NTT-FLAG
PLU-1-FLAG
Count
Distance from FeatureStart
(g)
FLAG-H3K4me3 peak overlap genome-wide
NTT-FLAG
PLU-1-FLAG
2.6%
97.4%
3.2%
96.8%
Overlapping
Non-Overlapping
