# Supplementary material for: The Catalytically Inactive KDM5B-NTT Isoform Associates with Chromatin and Increases H3K4 Trimethylation in Breast Cancer Cells
Source: Int J Mol Sci. 2026 Sep 30;27(19):8774. doi: 10.3390/ijms27198774 (PMC13664838; doi:10.3390/ijms27198774)
Supplement: Supplementary file 1 [file ijms-27-08774-s001.zip › FigureS2.pdf]

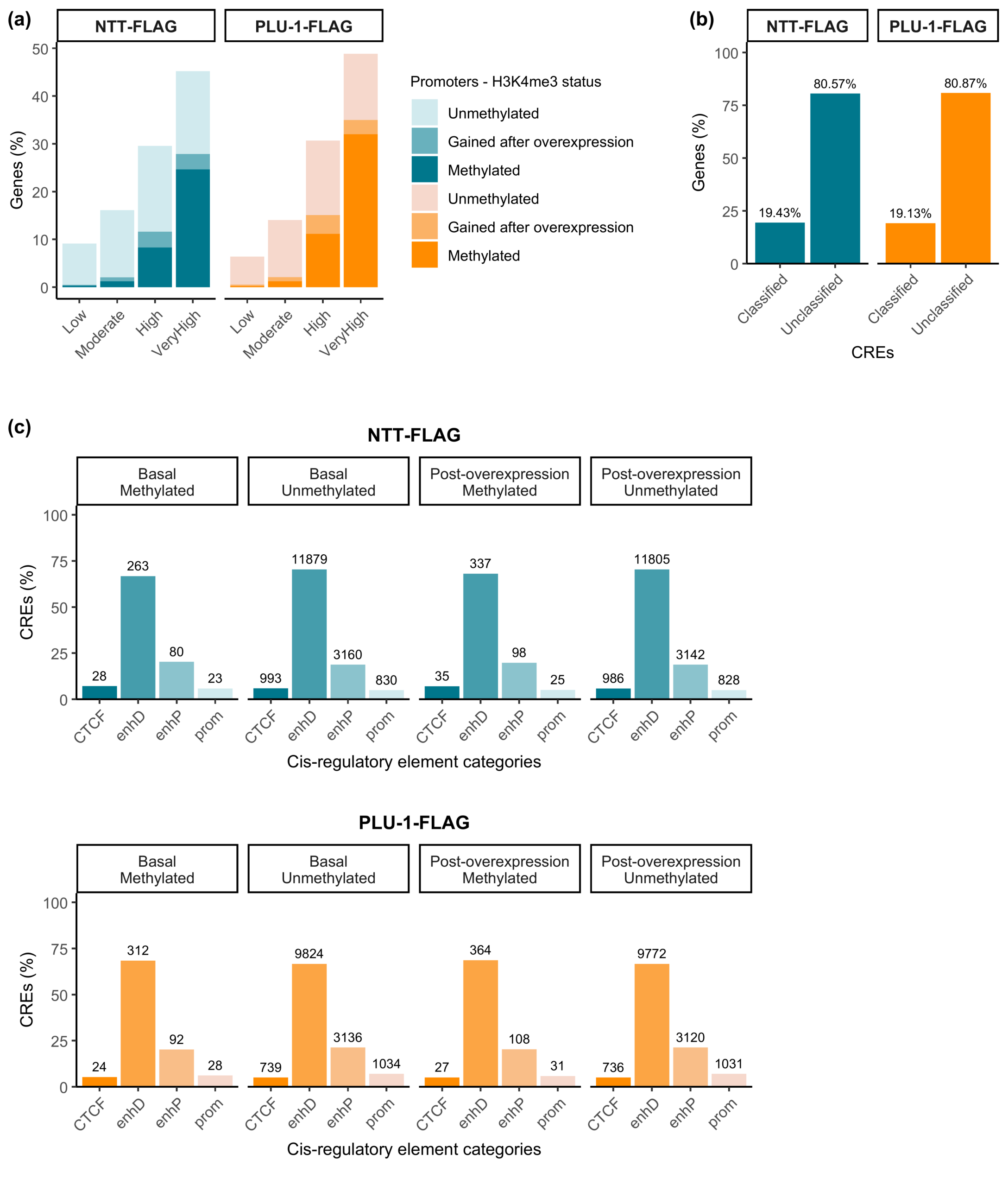
(a)
NTT-FLAG
PLU-1-FLAG
Genes (%)
Low
Moderate
High
VeryHigh
Promoters - H3K4me3 status
Unmethylated
Gained after overexpression
Methylated
Unmethylated
Gained after overexpression
Methylated
(b)
NTT-FLAG
PLU-1-FLAG
Genes (%)
19.43%
80.57%
19.13%
80.87%
Classified
Unclassified
CREs
(c)
NTT-FLAG
Basal Methylated
Basal Unmethylated
Post-overexpression Methylated
Post-overexpression Unmethylated
CREs (%)
28
263
80
23
993
11879
3160
830
35
337
98
25
986
11805
3142
828
CTCF
enhD
enhP
prom
Cis-regulatory element categories
PLU-1-FLAG
Basal Methylated
Basal Unmethylated
Post-overexpression Methylated
Post-overexpression Unmethylated
CREs (%)
24
312
92
28
739
9824
3136
1034
27
364
108
31
736
9772
3120
1031
CTCF
enhD
enhP
prom
Cis-regulatory element categories
