# Supplementary material for: The Catalytically Inactive KDM5B-NTT Isoform Associates with Chromatin and Increases H3K4 Trimethylation in Breast Cancer Cells
Source: Int J Mol Sci. 2026 Sep 30;27(19):8774. doi: 10.3390/ijms27198774 (PMC13664838; doi:10.3390/ijms27198774)
Supplement: Supplementary file 1 [file ijms-27-08774-s001.zip › FigureS3.pdf]

(a)

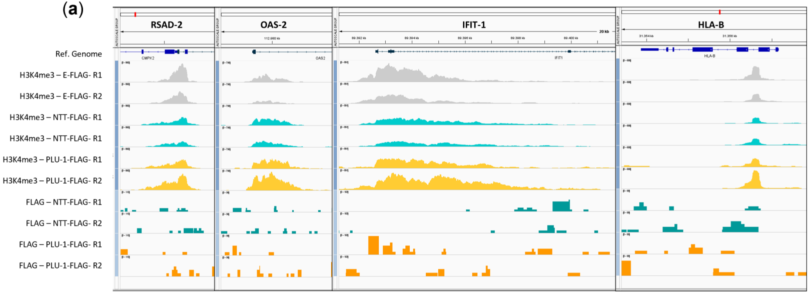
RSAD-2
OAS-2
IFIT-1
HLA-B
Ref. Genome
H3K4me3 – E-FLAG- R1
H3K4me3 – E-FLAG- R2
H3K4me3 – NTT-FLAG- R1
H3K4me3 – NTT-FLAG- R1
H3K4me3 – PLU-1-FLAG- R1
H3K4me3 – PLU-1-FLAG- R2
FLAG – NTT-FLAG- R1
FLAG – NTT-FLAG- R2
FLAG – PLU-1-FLAG- R1
FLAG – PLU-1-FLAG- R2

(b)

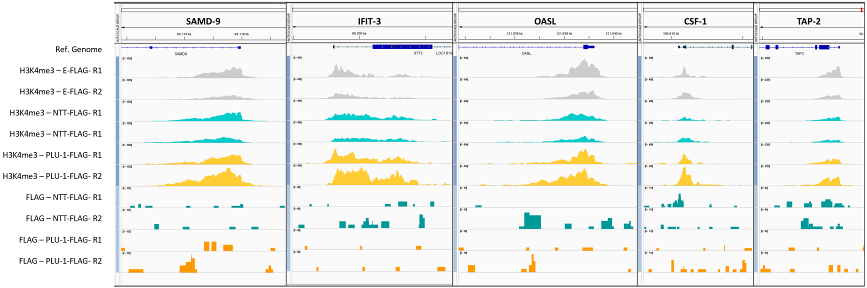
SAMD-9
IFIT-3
OASL
CSF-1
TAP-2
Ref. Genome
H3K4me3 – E-FLAG- R1
H3K4me3 – E-FLAG- R2
H3K4me3 – NTT-FLAG- R1
H3K4me3 – NTT-FLAG- R1
H3K4me3 – PLU-1-FLAG- R1
H3K4me3 – PLU-1-FLAG- R2
FLAG – NTT-FLAG- R1
FLAG – NTT-FLAG- R2
FLAG – PLU-1-FLAG- R1
FLAG – PLU-1-FLAG- R2
