# Supplementary material for: The Catalytically Inactive KDM5B-NTT Isoform Associates with Chromatin and Increases H3K4 Trimethylation in Breast Cancer Cells
Source: Int J Mol Sci. 2026 Sep 30;27(19):8774. doi: 10.3390/ijms27198774 (PMC13664838; doi:10.3390/ijms27198774)
Supplement: Supplementary file 1 [file ijms-27-08774-s001.zip › FigureS4.pdf]

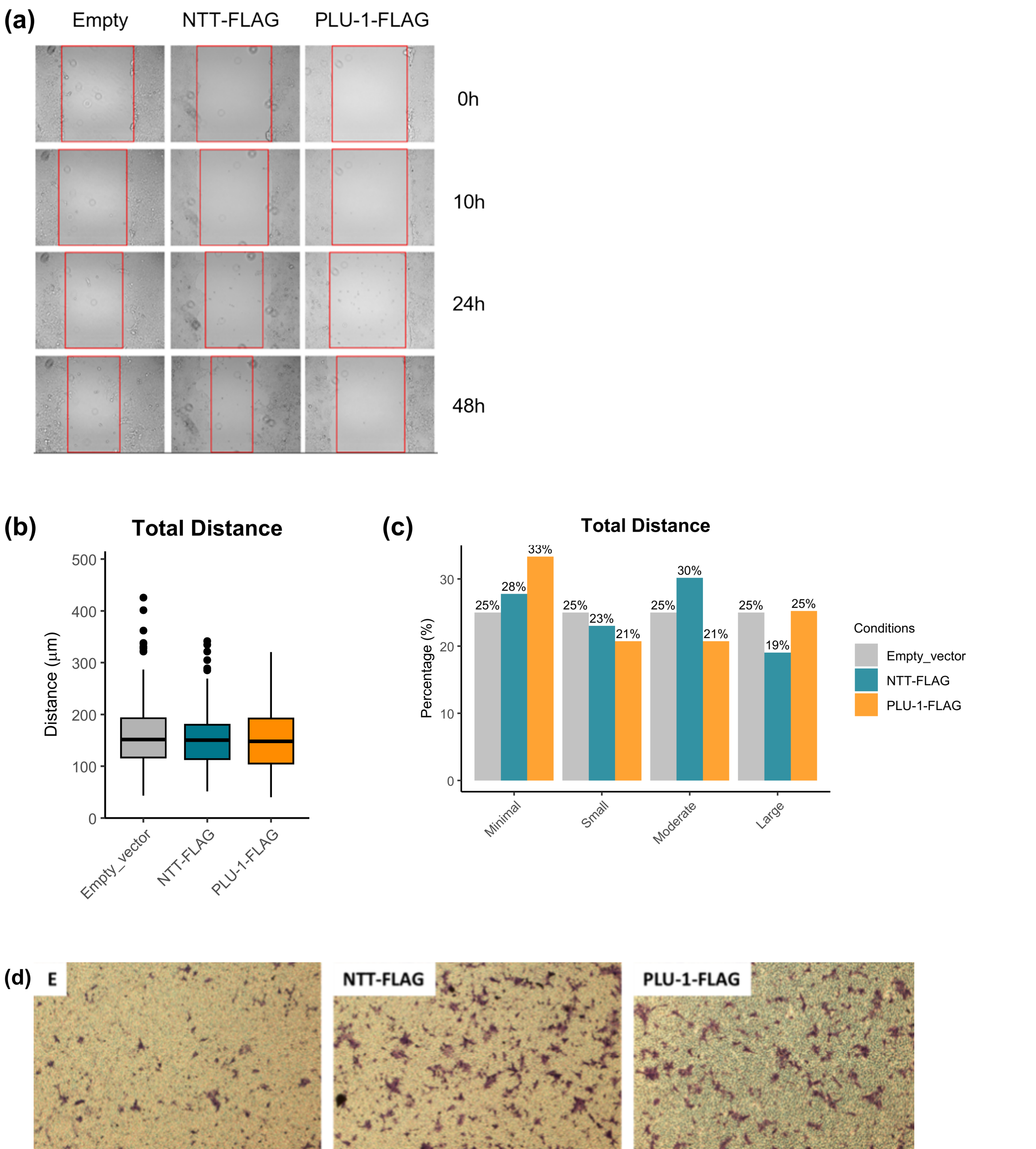

(a)
Empty
NTT-FLAG
PLU-1-FLAG
0h
10h
24h
48h
(b)
Total Distance
Distance (μm)
0
100
200
300
400
500
Empty_vector
NTT-FLAG
PLU-1-FLAG
(c)
Total Distance
Percentage (%)
0
10
20
30
Minimal
Small
Moderate
Large
25%
28%
33%
25%
23%
21%
25%
30%
21%
25%
19%
25%
Conditions
Empty_vector
NTT-FLAG
PLU-1-FLAG
(d)
E
NTT-FLAG
PLU-1-FLAG
