# Supplementary material for: The Catalytically Inactive KDM5B-NTT Isoform Associates with Chromatin and Increases H3K4 Trimethylation in Breast Cancer Cells
Source: Int J Mol Sci. 2026 Sep 30;27(19):8774. doi: 10.3390/ijms27198774 (PMC13664838; doi:10.3390/ijms27198774)
Supplement: Supplementary file 1 [file ijms-27-08774-s001.zip › Supplementary_files_revised.pdf]

Supplementary figures

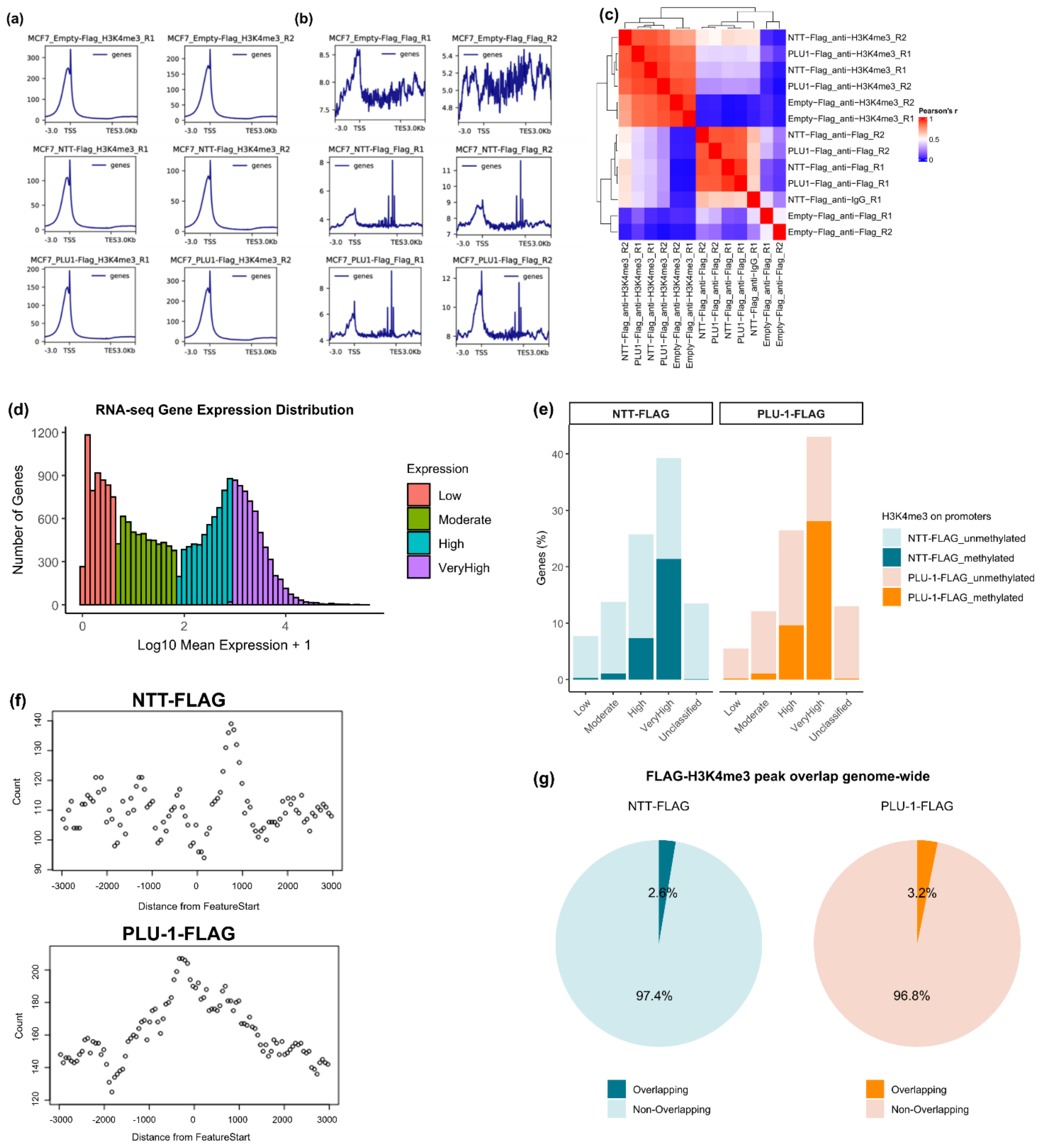

Figure S1. Quality control and epigenomic characterization of CUT&RUN data in NTT-FLAG and PLU-1-FLAG overexpressing cells. (**a-b**) Distribution of signal starting from 3 Kb upstream of the transcription start site (TSS), along the gene body to 3 Kb downstream of the transcription end site (TES). Graphs display signals obtained across all three conditions (Empty, NTT-FLAG, PLU-1 FLAG) in both biological replicates (R1, R2) for (**a**) H3K4me3 marks and (**b**) anti-FLAG; (**c**) Sample-to-sample correlation heatmap displaying the Pearson's R correlation coefficients between CUT&RUN samples, indicating inter-sample relationship strength (blue: coeff. = 0; red: coeff. = 1); (**d**) Distribution of mean gene expression derived from RNA-seq data (NTT-FLAG overexpression vs. Empty), categorized by quartiles into four transcriptional classes (Low, Moderate, High, Very High); (**e**) Promoter methylation status of FLAG-bound genes across distinct transcriptional classes. Dark shades indicate promoters harbouring at least one H3K4me3 peak, while light shades

represent the percentage of unmethylated promoters (teal for NTT-FLAG condition and orange for PLU-1-FLAG); (**f**) Distribution of aggregated peak counts around the TSS. Graphs display the second biological replicate for each condition as a representative example; (**g**) Pie charts representing the genome-wide distribution of all FLAG peaks, independent of functional genomic annotations. Dark shades denote overlap (direct or proximal) between FLAG-interacting regions and basal H3K4me3 marks (Empty vector), while light shades indicate regions lacking overlap.

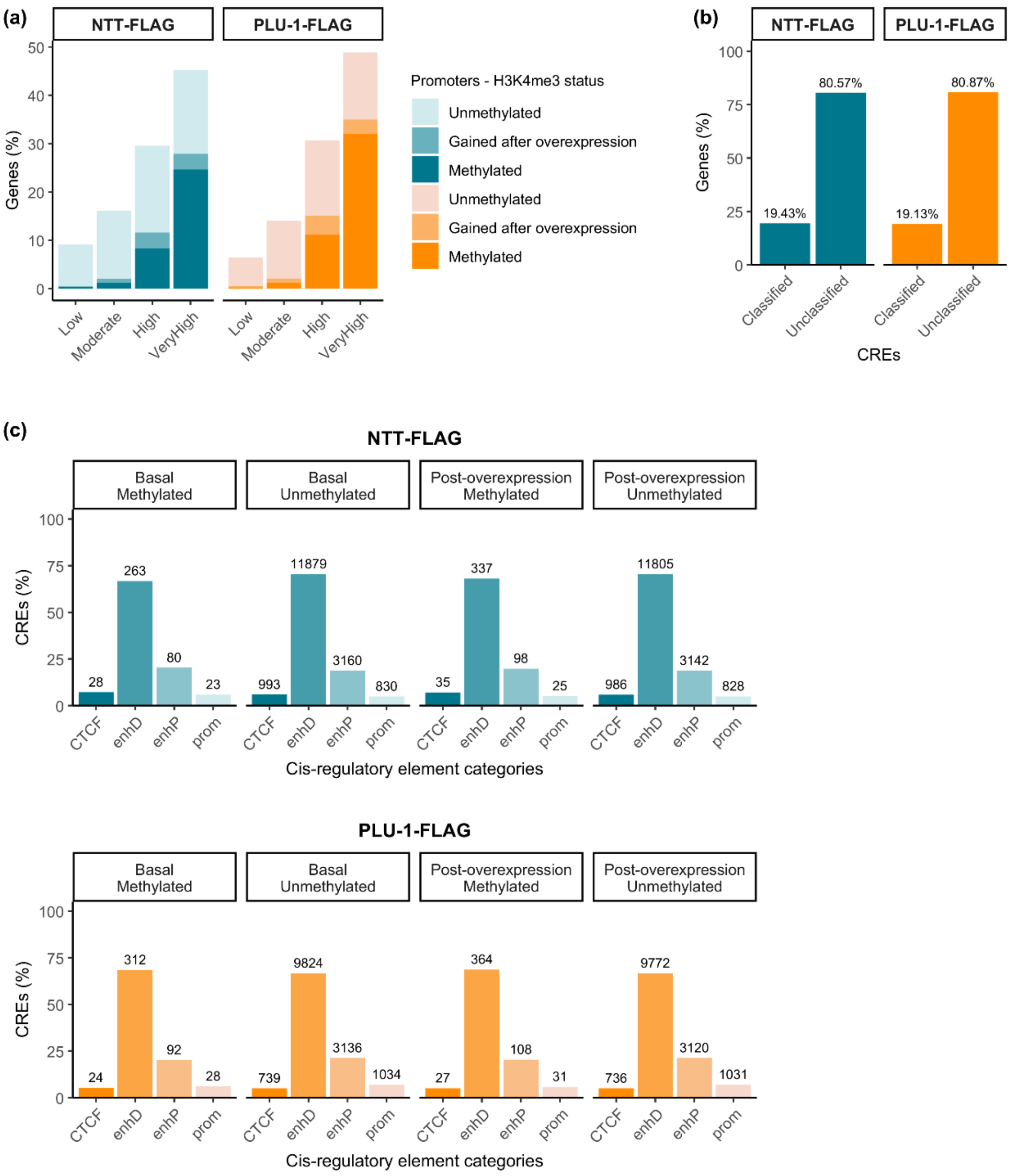

Figure S2. H3K4me3 status at FLAG-interacting promoters and cis-regulatory elements upon overexpression of KDM5B isoforms. This figure extends the characterization of NTT-FLAG and PLU-1-FLAG binding sites by detailing the H3K4me3 methylation state at promoters across transcriptional classes, and by describing the distribution and trimethylation status of isoform binding sites within ENCODE-defined cCREs. (**a**) H3K4me3 at gene promoters harbouring NTT-FLAG (teal) or PLU-1-FLAG (orange) binding sites, stratified by transcriptional class. Dark shades represent promoters that are consistently methylated both under basal conditions (empty vector-treated cells) and upon overexpression; intermediate shades indicate promoters that acquire methylation only after overexpression; light shades indicate promoters that remain unmethylated under both conditions; (**b**) Proportion of the genome classified as candidate cis-regulatory elements (cCREs) according to ENCODE annotation, alongside regions not falling within any defined cCRE category, shown for NTT-FLAG (teal) and PLU-1-FLAG (orange) binding sites; (**c**) H3K4 status of cCREs harbouring FLAG binding sites, before and after overexpression of NTT-FLAG (teal) or PLU-1-FLAG (orange). The y-axis represents the percentage of regions belonging to each regulatory category; the number of sequences mapping to each cCRE class is indicated within each bar. Individual panels distinguish the trimethylation status under basal conditions (basal state) from that observed following overexpression.

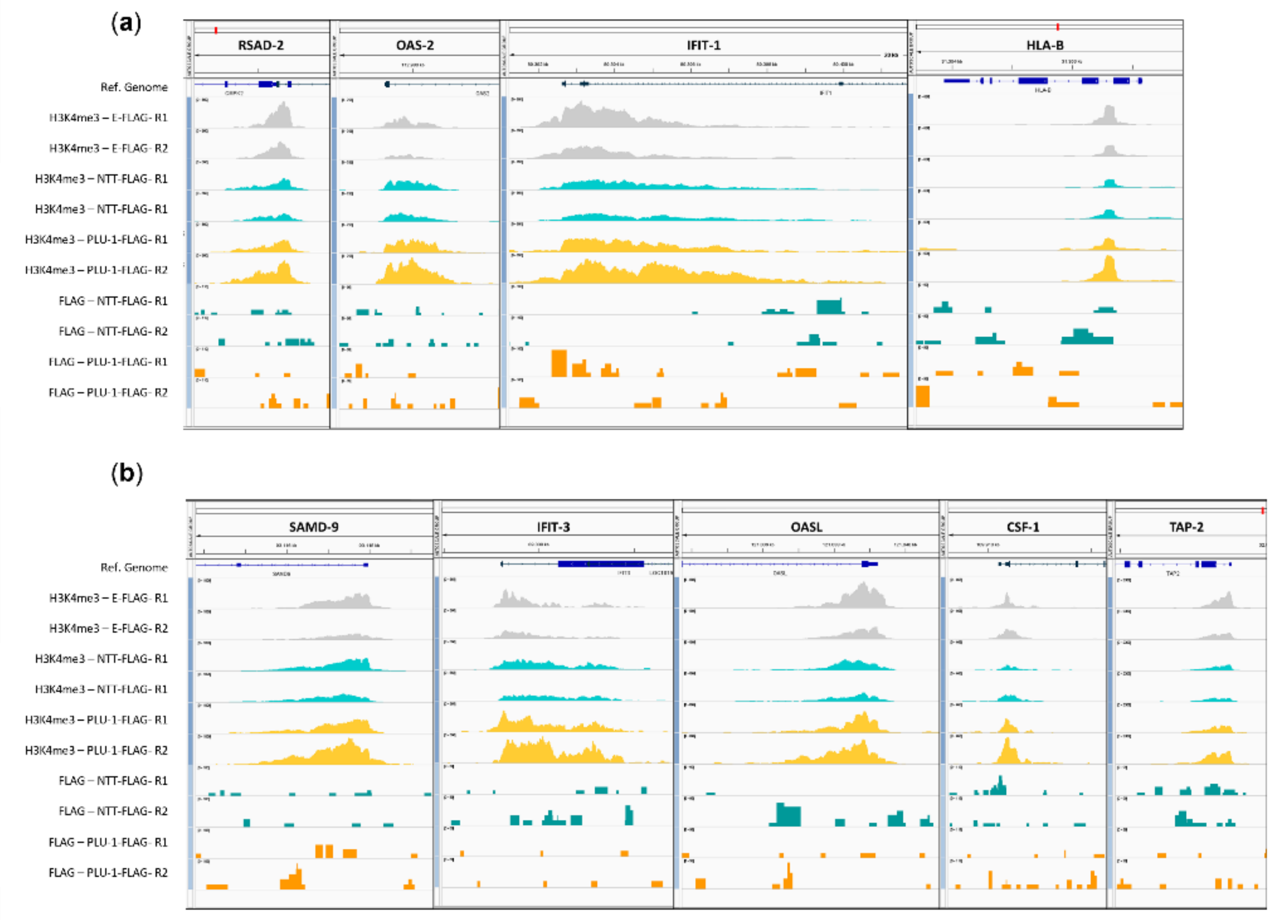

Figure S3. H3K4me3 and FLAG binding profiles at promoters of differentially expressed genes associated with NTT-FLAG and PLU-1-FLAG. This figure displays Integrative Genomics Viewer (IGV) tracks from CUT&RUN experiments, illustrating H3K4me3 and FLAG occupancy at the promoters of selected DEGs. (**a**) IGV tracks for four DEGs bound exclusively by NTT-FLAG. (**b**) IGV tracks for five DEGs bound by both NTT-FLAG and PLU-1-FLAG. In both panels, tracks are displayed from top to bottom as follows: gene annotation and reference genome; H3K4me3 signal in empty-vector conditions (grey, replicates 1 and 2); H3K4me3 signal in NTT-FLAG overexpressing cells (light teal, replicates 1 and 2); H3K4me3 signal in PLU-1-FLAG condition (light orange, replicates 1 and 2); anti-FLAG signal in NTT-FLAG condition (teal, replicates 1 and 2); anti-FLAG signal in PLU-1-FLAG overexpressing cells (orange, replicates 1 and 2).

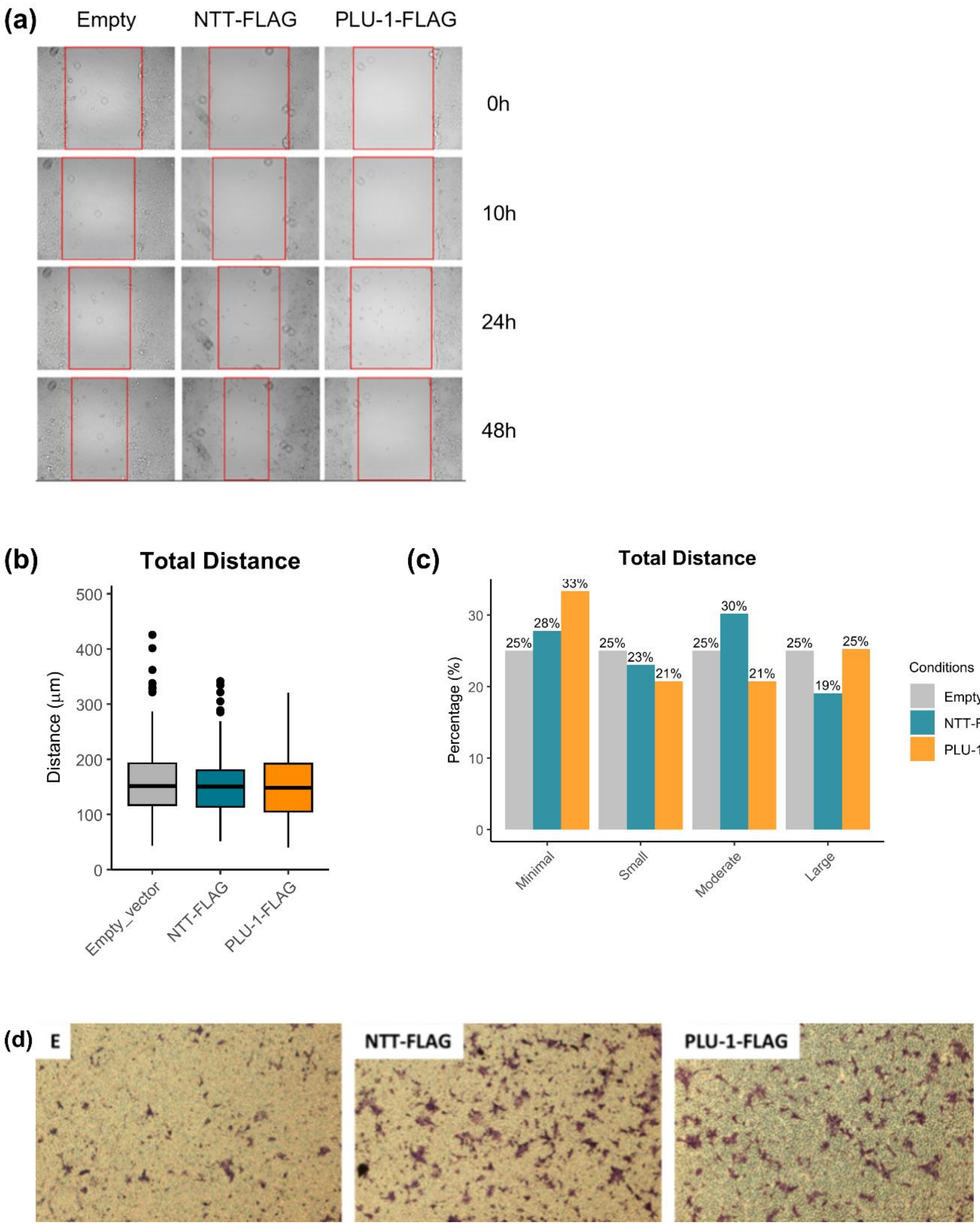

Figure S4. Morphological assessment of migration and invasion in MCF7 cells overexpressing KDM5B isoforms. Representative images and additional parameters referred to live imaging migration corroborate the divergent migratory and invasive phenotypes induced by NTT-FLAG and PLU-1-FLAG overexpression. (**a**) Representative brightfield images of the wound healing assay in cells overexpressing Empty vector, NTT-FLAG, or PLU-1-FLAG, acquired at 0, 10, 24, and 48 hours post-wound. Dashed lines delineate the wound margins at each time point across the indicated conditions. (**b**) Total distance travelled of cells, measured by live-imaging migration assay (biological triplicate). Statistical comparisons were performed using Dunn's test. (**c**) Distribution of MCF7 cells across migration classes defined by total distance travelled under the indicated conditions. (**d**) Representative microscopy images (10× magnification) of cells stained with crystal violet following invasion through a Geltrex-coated Transwell membrane, shown for each indicated condition.

Table S1. Gene Set Enrichment Analysis (GSEA) of biological processes associated with genes harboring NTT-FLAG and PLU-1-FLAG binding sites. In the table, only the subgroup of DEGs (previously described in Di Nisio et al. [33]) selected for the presence of NTT-FLAG or PLU-1-FLAG binding sites is reported.

Biological Process	*N. of genes*
HALLMARK_INTERFERON_GAMMA_RESPONSE	21
HALLMARK_INTERFERON_ALPHA_RESPONSE	17
HALLMARK_ALLOGRAFT_REJECTION	10
HALLMARK_TNFA_SIGNALING_VIA_NFKB6	10
HALLMARK_COMPLEMENT	9
HALLMARK_COAGULATION	8
HALLMARK_EPITHELIAL_MESENCHYMAL_TRANSITION	7
HALLMARK_IL6_JAK_STAT3_SIGNALING	7
HALLMARK_UV_RESPONSE_DN	7
HALLMARK_INFLAMMATORY_RESPONSE	6
HALLMARK_MYOGENESIS	6
HALLMARK_APOPTOSIS	5
HALLMARK_HYPOXIA	5
HALLMARK_KRAS_SIGNALING_DN	5
HALLMARK_XENOBIOTIC_METABOLISM	5
HALLMARK_APICAL_JUNCTION	4
HALLMARK_IL2_STAT5_SIGNALING	4
HALLMARK_KRAS_SIGNALING_UP	4

Table S2. List of primers used for RT-qPCR analyses.

Target	Sense	Sequence (5’-3’)
**PLAUR**	Forward	TTGTGGGAAGAAGGAGAAGAGC
**PLAUR**	Reverse	TAACGGCTTCGGGAATAGGTG
**CSF1**	Forward	ATGGAGACCTCGTGCCAAAT
**CSF1**	Reverse	AGGCCTTGTCATGCTCTTCA
**ACTIN**	Forward	TCGTGCGTGACATTAAGGAG
**ACTIN**	Reverse	AGGAAGGAAGGCTGGAAGAG
